# The Episodic Prototypes Model (EPM): On the nature and genesis of facial representations

**DOI:** 10.1177/20416695211054105

**Published:** 2021-10-29

**Authors:** Tobias Matthias Schneider, Claus-Christian Carbon

**Affiliations:** Department of General Psychology and Methodology, University of Bamberg, Bavaria, Germany; Research Group EPÆG (Ergonomics, Psychological Æsthetics, Gestalt), Bamberg, Germany; Bamberg Graduate School of Affective and Cognitive Sciences (BaGrACS), Bamberg, Germany

**Keywords:** facial representations, face prototypes, face-specific processing, adaptation, face learning, development, updating, life episode

## Abstract

Faces undergo massive changes over time and life events. We need a mental representation which is flexible enough to cope with the existing visual varieties, but which is also stable enough to be the basis for valid recognition. Two main theoretical frameworks exist to describe facial representations: prototype models assuming one central item comprising all visual experiences of a face, and exemplar models assuming single representations of each visual experience of a face. We introduce a much more ecological valid model dealing with episodic prototypes (the Episodic Prototypes Model—EPM), where faces are represented by a low number of prototypes that refer to specific Episodes of Life (EoL, e.g., early adulthood, mature age) during which the facial appearance shows only moderate variation. Such an episodic view of mental representation allows for efficient storage, as the number of needed prototypes is relatively low, and it allows for the needed variation within a prototype that keeps the everyday and steadily ongoing changes across a certain period of time. Studies 1–3 provide evidence that facial representations are highly dependent on temporal aspects which is in accord with EoL, and that individual learning history generates the structure and content of respective prototypes. In Study 4, we used implicit measures (RT) in a face verification task to investigate the postulated power of the EPM. We could demonstrate that episodic prototypes clearly outperformed visual depictions of exhaustive prototypes, supporting the general idea of our approach.

## 1. Introduction

Face researchers mainly concentrate on the perception of faces and how we can recognize them (see, e.g., Akselrod-Ballin & Ullman, 2008; Burton et al., 2005; Carbon, 2011; Gao & Wilson, 2014; Jenkins & Burton, 2008; Mileva et al., 2020; Patterson & Baddeley, 1977; Schneider & Carbon, 2017b). A topic which is much less investigated and systematized is how facial representations and prototypes are generated, and on which experiences they are established.

**Figure 1.**
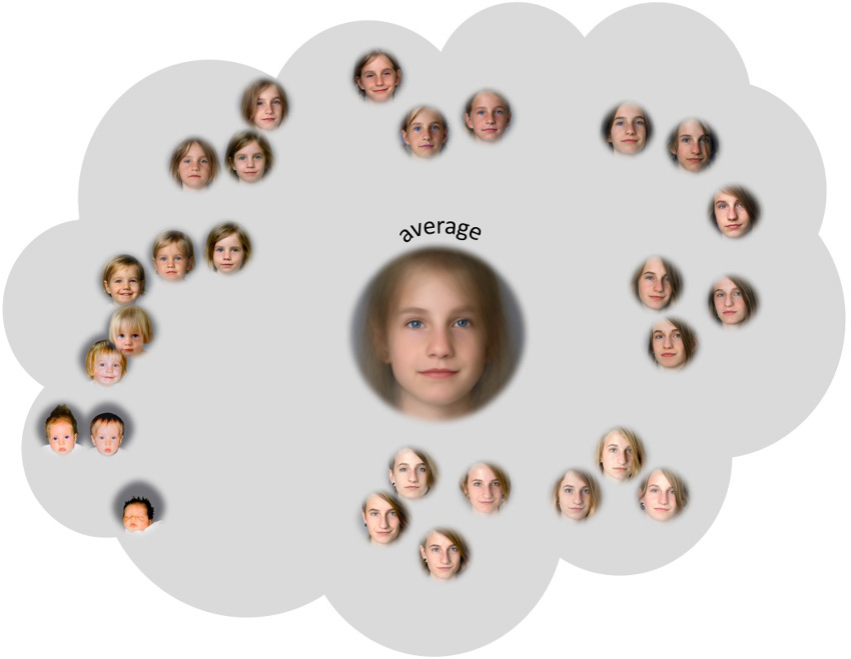
An exemplary illustration of a “classical” individual-level face-space: facial exemplars are encoded as points in this multidimensional space and their distances correspond to the perceived (dis-)similarity. In classical individual-level face-space models (this is the term we will use to refer to face-space models for the mental representations of facial depictions of individual persons), the average prototype which is usually described as the centroid of the face-space consists of the sum of all exemplars and is assumed to be the most typical presentation. © by Michael Langoth, who allowed us to use these pictures for our research and the corresponding publication—see authors’ note.

With regard to prototypes in general, recognized scientific theories such as the ‘recognition by prototypes’ theory (Basri, 1996) define prototypes as averages of given exemplars or of their principal components (e.g., Burton et al., 2005; Gao and Wilson, 2014). Further theories (see, e.g., Busey, 1998; Valentine, 1991; Valentine and Endo, 1992) postulate a so-called *multidimensional face-space model*. It is important to mention that such a face-space could mainly be interpreted in two ways. First, population-level face-spaces (referring to face-spaces across different identities) (e.g., Busey, 1998; Valentine and Endo, 1992). Here, the face-discriminating dimensions refer to, e.g., ethnicity, distinctiveness, etc. Second, individual-level face-spaces: given a certain familiar facial identity, the respective prototype (most typical face) provides the centroid of this face-space, whereas less typical exemplars of the identity are less densely clustered in the periphery (we will refer to this interpretation for the present study). All unique exemplars of a face are encoded as points in this *n*-dimensional space along face-discriminating dimensions such as facial expression, age, etc. The distances between two points are analogous to the dis-similarity between the respective exemplars (see Figure 1). It is assumed that this face-space corresponds to one's facial representation of this particular identity in the associative network (see e.g., Benson and Perrett, 1993; Busey, 1998; Reinitz et al., 1992; Webster et al., 2004; Webster & MacLeod, 2011).

These intuitive face-space models describing the cognitive organization of faces in our mental representation are indeed supported by findings of empirical studies on mental representations. With respect to the so-called *prototype-distance model* or *central tendency model* (see e.g., Franks and Bransford, 1971), prototypes could be described as *abstractions* of a certain concept formulated by *averaging characteristic features*. Moreover, neither the prototype nor its features are necessarily experienced in the past, since the prototype and its components emerge from an *averaging process* based on direct experience with the exemplars. Accordingly, participants tend to recognize an averaged dot pattern (prototype) with higher confidence even if they did not see this prototype in the preceding learning session—the so-called “prototype effect” (Posner & Keele, 1968, 1970). This central tendency model was further extended by Reed (1972), who suggested that the prototype is altered by each experience with a new object, with the influence of individual stimuli decreasing as the total number of experienced objects increases. Similarly, Faerber et al. (2016) recently postulated the so-called *recalibration hypothesis* wherein the process of updating a prototype ( = familiarization with a given face) is associated with an increasing level of typicality. This is based on the idea of “resetting” and “readjustment” mechanisms propagated by Carbon et al. (2007) for explaining long-term adaptation effects.

Alternative models like the *exemplar-based representation models* follow a completely different conceptual idea. They postulate that a certain prototype does *not necessarily* exist (consisting of an abstraction of *all* characteristic attributes of the respective concept). Instead, the mental representation consists of the entirety of *all* experienced objects of that concept (Brooks, 1978) as different and explicit “exemplars”. Hintzman (1984, 1986) suggested an implementation of the *exemplar-based* model idea, which he also empirically investigated—the so-called *MINERVA 2 model*: Given a *new* object that should be identified, the *similarity* to all exemplars stored in memory is calculated in a first step. Then in a second step, the features of the stored exemplars are weighted with these values of similarity. The features of exemplars with higher similarity are weighted higher, resulting in a so-called “*echo”* as a next step. The simultaneous activation of all exemplars by a new object produces a single echo. The echo's intensity is the sum of the activation levels of all represented exemplars. This echo is calculated by adding up all weighted features of inherent exemplars relating to a concept. The new object is then matched to the concept with the strongest echo. These exemplar-based models seem to have some interesting advantages over prototype models: Regarding facial processing, recent studies investigated the highly related process of how an unfamiliar face becomes familiar and revealed that learning a new face involves an abstraction of the *variability* of different images belonging to the very same person's face (see, e.g., Burton et al., 2016; Kramer et al., 2017; Menon et al., 2015; Ritchie et al., 2018; Ritchie & Burton, 2017; Young & Burton, 2017). The authors were able to show that faces vary in systematic ways, and that this variability is somehow “idiosyncratic”, for instance, facial expression and hairstyle. Accordingly, they suggested that the process of learning a new face is based on learning how that face varies—precisely this might be the only straightforward approach to preventing rigid or “iconic” facial representations (Carbon, 2008). Despite the fact that the variability of outward facial appearances plays a major role in this approach, we are not offered any information about *how* these faces are mentally represented (e.g., more *exemplar-based* vs. more *prototype-based*). Actually, even how variability emerges and what kind of variability is of particular interest is not specified within these approaches—in fact, even the most varying pictures which are utilized within these very promising approaches originate from a rather limited time frame.

**Figure 2.**
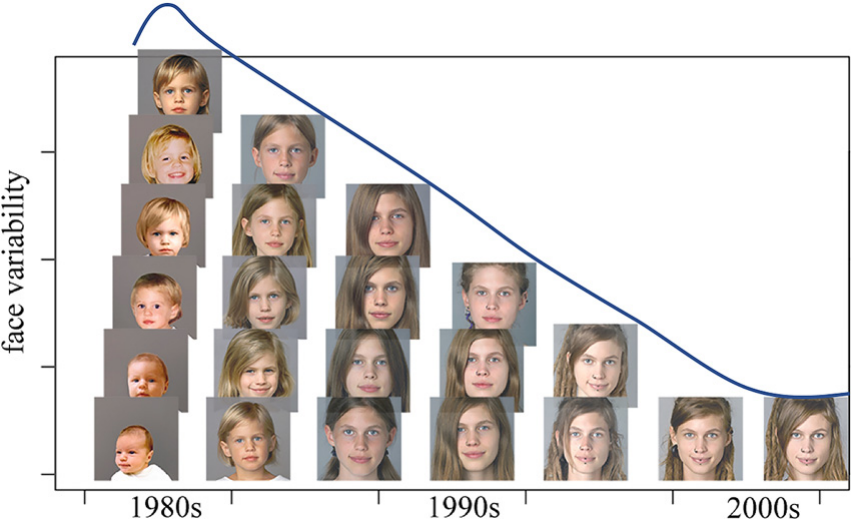
Demonstration of the development of facial characteristics across a span of around 30 years of the very same person's life. Typically, the development of facial characteristics is stronger in the early years and decreases with advancing age. © by Michael Langoth, who allowed us to use these pictures for our research and the corresponding publication —see authors’ note.

One major source of variability in outward facial appearances is *aging* (Davies et al., 1981; Johnston et al., 1997). Focusing on aging factors will allow us to test explicitly how a face can still be efficiently recognized when the target face has changed since the last encounter due to the effects of aging (Carbon, 2008). This is exactly what is required in everyday life where we have to face such effects. It is quite striking how much a face changes during a lifetime (Carbon, 2008)—especially in the first years of life, but also continuously throughout later life (see Figure 2). In everyday life, we learn new faces by experiencing the respective person over a limited stretch of some years, a very long time with temporal interruptions, or even continuously across an entire lifetime. On the basis of such learning histories, a reliable recognition mechanism has to cope with these varieties (see, e.g., Matthews et al., 2018). From an evolutionary perspective, it seems important to focus on the more recent experiences as the past is past, and so the representations of nearer-to-present experiences are probably better indicators for successful recognition in the future. We can argue even more drastically: from an evolutionary perspective, it is relatively unclear what the benefit of recognizing past pictures is at all as there is no such external memory as photographs in the realm of evolution. This bias towards the relevance of more recent information could be addressed by *weighting* the temporal aspects of (visual) experiences—this mechanism can be seen with the so-called figural after-effect, an adaptation mechanism towards recent experiences with the respective individual (see, e.g., Carbon and Ditye, 2011, 2012)—see Strobach and Carbon (2013) for a review on several adaptation factors. Accordingly, it seems obvious that a rigidly averaged facial depiction of a particular person over a large span of time (e.g., in the sense of an *exhaustive* prototype across several decades or even a full lifetime) certainly does not optimally correspond to the actual mental representation of the respective face. More importantly, the actual existence of such exhaustive representations seems to be idealistic since we will not find a person that has had equal exposure to *all* instances of facial exemplars across the whole lifespan. Aside from the fact that most past research never explicitly postulated such an exhaustive prototype it is at least compatible with such averaging approaches since a prototype consisting of all possible (or at least as many as possible) instances of an identity may be most likely to get recognized later on (see Burt and Perrett, 1997; Burton et al., 2005; Jenkins and Burton, 2008; Posner and Keele, 1968, 1970). However, with facial development over a more extended period of time, the idiosyncratic variability increases and the informative content of an exhaustive prototype decreases. Accordingly, this exhaustive prototype is relatively unspecific for the recent appearance of a given face.

Current representation models mostly neglect the aging factor. However, some very few studies which considered age, *explicitly* encoded it as a factor or, in the case of the multidimensional face-space, added an extra *age dimension* (e.g., Busey, 1998; Johnston et al., 1997); such approaches handle age as a kind of abstracted individual-independent quale, operationalized by aging effects across *different* individuals. It is clear that such an abstracted age dimension is highly implausible, because aging is typically a much more idiosyncratic factor which is confounded by the alteration of skin, with the adding of wrinkles and the overall morphological change towards a more asymmetric outward appearance (see e.g., Azizi et al., 1988; Matts & Fink, 2010; Schneider et al., 2012). We have called this age dimension “abstracted” as this approach makes us believe that age can be abstracted from other facial dimensions; but this would imply that age is just an additional though not interactive factor—interactive based on idiosyncratic developments. Actually, an abstracted age view would obviously lose a lot of explanatory variance of individual outward appearances of faces and thus would operate sub-optimally. Only preliminary research (Mileva et al., 2020) exists proposing that individual faces consist of physical commonalities across a lifespan. Importantly, the authors revealed that participants who learned clusters of faces *within a time period* were able to cope with recognition tasks beyond that of identifying faces representing other periods of a life. Besides the fact that this robust recognition performance may break down at a certain level (e.g., after 30 years), these findings underline the importance of highlighting *age* as a major source of face variability. These results typically rely on rather simulation-based studies than on actual participant-based studies which reflect the more naturalistic way of learning faces. Mileva et al. (2020) used sets of faces with no time gaps vs. 20-year time gaps vs. 40-year time gaps to test the robustness and validity of such clusters with respect to face recognition across a lifespan. However, the specific mechanism of *how* we learn faces across a lifespan and *how* such clusters are established remain unclear. Furthermore, we do not know whether the used sets actually correspond to a facial representation of a presented identity and whether it is more likely that such clusters are individually *idiosyncratic* of each identity.

**Figure 3.**
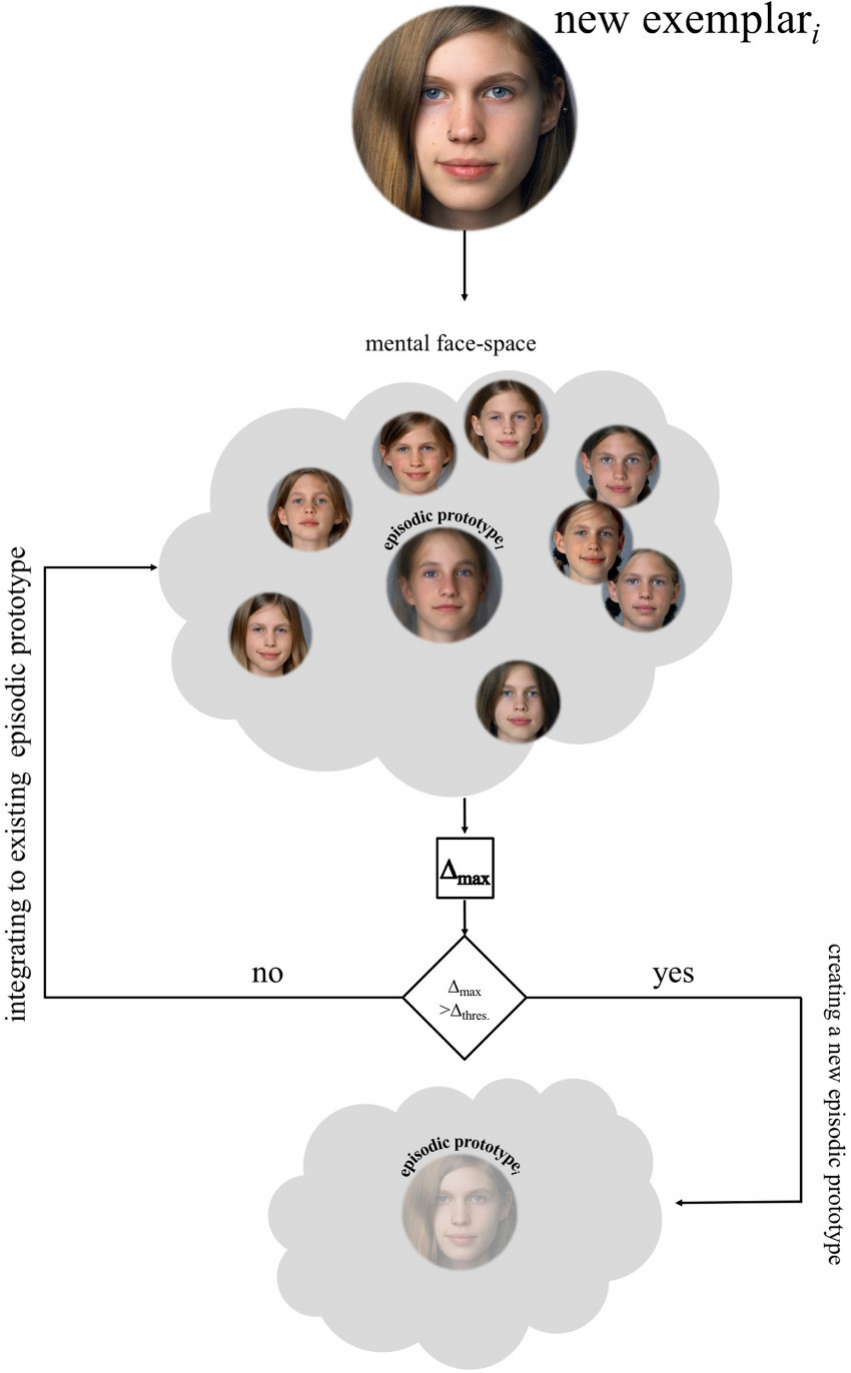
The genesis of “episodic prototypes”. These episodes refer to best representations of certain EoL (e.g., a “*baby episode*”, a “*youngster episode*”, an “*aged episode*” etc.). Given that a new facial exemplar *i* is experienced, a maximum delta has to be calculated. This value (maximum delta) refers to the maximum dis-similarity of a new experienced face to the existing episodic prototype and its exemplars. If the new face is too far away from the existing exemplars of the corresponding episodic prototype*_1_* (upper cloud), a new prototype has to be generated (lower cloud); otherwise, this exemplar is integrated into the existing face-space. This refers to the operation of comparing the maximum delta with a threshold-delta that is pre-fixed. Note that this illustration is an exemplary demonstration of the genesis, starting with a single pre-existing episodic prototype (prototype*_1_*). This integrating or generating process is ongoing, and so every new experienced face will be matched to all existing episodic prototypes*_i_*.

A potential solution to this problem could be to propose prototypes that represent outward facial appearances from specific time periods in an individual human life, e.g., a “*baby episode*”, a “*youngster episode*”, an “*aged episode*” etc. We would like to call such representations “episodic prototypes” in the following (see Carbon, 2009). Episodes of this kind refer to distinctive sub-prototypical representations. If a new exemplar of a face is too far away from the centroid of such an episodic prototype, a new prototype has to be generated (see an illustration of such a process in Figure 3). This proposed process means that one person's outward facial appearance is potentially represented by multiple episodic prototypes which are semantically linked together. In many cases, this will mean that some episodic prototypes will be quite independently stored without such a semantic link. For instance, if we know an actor from a certain time of her or his career only, and if we then encounter the very same actor in a film of a different epoch showing a very different outward facial appearance, we might not get this link as we might solely rely on visual cues. There is some evidence that we might identify this actor in a face recognition task after years (see e.g., Bruck et al., 1991), however, it might take us longer in terms of a response since development / change of facial characteristics might be quite substantial across different episodes of his life. These episodes reflect certain time spans or *Episodes of Life* (EoL). As our model defines episodes according to aspects of similarity, these episodes will evidently mimic specific EoL, although they might differ from the typical episodic constructs assumed by developmental psychology.

**Figure 4.**
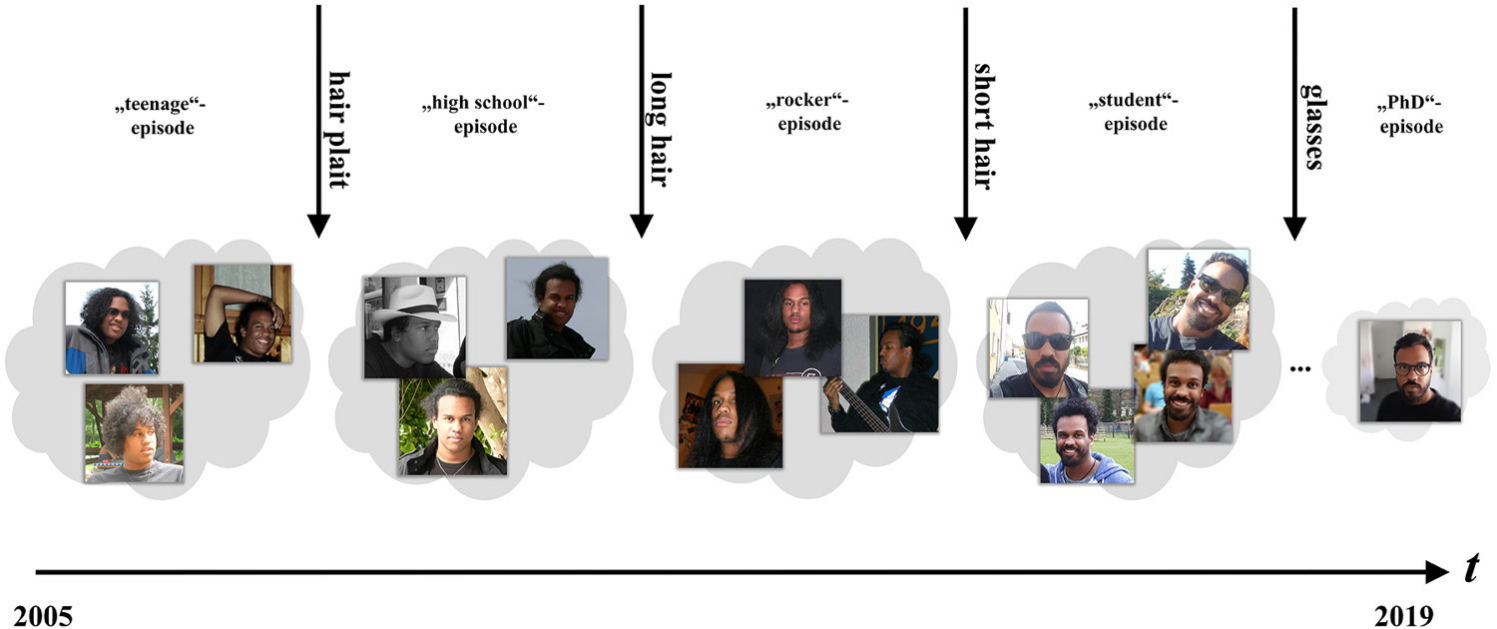
Episodic prototypes are based on typical and distinct EoL. When past experiences with a face are too different to present experiences (e.g., due to a very new hairstyle), a new prototype has to be generated in order to limit the variance of the respective exemplars.

What, however, determines whether a new episodic prototype has to be generated and why should we have multiple (episodic) prototypes of a certain identity? Our postulated approach is inspired by research from the field of human memory and cognition: so-called “generative memory models” (e.g., Daw and Courville, 2008; Yu and Dayan, 2005) which postulate that environments tend to change slowly over time, but suddenly jump to a completely different “mode” which requires the generation of a new memory trace (Gershman et al., 2014; Gershman et al., 2017). Gershman et al. (2014) provided an example of such a generative memory model: Usually, while the temperature within different parts of a building may fluctuate rather slowly, going outside is characterized by very different (though also slowly changing) temperatures than those that were in effect indoors. The authors further argue that we can recall the general temperature inside the building, and separately, the temperature outside of the building. Humans seem to have distinct and different memory traces according to multiple modes/states of a certain construct (in this example, temperature). Similarly, another important approach what supports our postulation is the so-called SUSTAIN model of categorial learning by Love et al. (2004): At the beginning of the mental categorization procedure, SUSTAIN suggests a simple category structure. If the model processes a sudden or surprising event so that the simple structure proves to be inappropriate, a new cluster is generated to represent this event. Other models like the predictive coding model follow a similar categorial abstraction approach to make inferences about future events (e.g., Spratling, 2016). Applying this to facial prototypes and representations, variables such as facial mass, which is highly correlated with body weight (e.g., Coetzee et al., 2009; Coetzee et al., 2010; Schneider et al., 2012; Schneider & Carbon, 2017b) or hair style usually changes rather slowly over the course of a human lifetime. For instance, the first author (Tobias) had an “Afro” in his teenage years; later, he had longer hair (in his “rocker”–phase), and at present, he has short hair and a beard (see Figure 4). When comparing these depictions, which are characteristic of episodes of his life, the changes seem to be quite dramatic, but occurred over a period of 10–15 years. However, in the case of a sudden change (shown in Figure 4: e.g., Tobias cut his hair short in 2015), there is a strong mismatch between past experiences of a given face and a new experience. By using a simple similarity approach, the model provides a very efficient coding that allows effective retrieval because an outward facial appearance which is too dissimilar to already existing episodic prototypes will automatically yield the generation and establishment of a new episodic prototype. Every prototype is inherently optimized by being composed of similar but not identical exemplars. This creates the required variety of exemplars that leads to reliable and fast recognition later on (e.g., Jenkins et al., 2011; Mileva et al., 2020; Ritchie and Burton, 2017). At the same time, as the formation of a new episodic prototype needs substantial deviation from already represented episodic prototypes, the number of such prototypes is delimited to a low number that prevents overloading the cognitive system.

The main aim of the present paper is to develop a new model for the genesis of facial prototypes and representations. This model will explicitly consider variations of face exemplars mainly caused by temporal factors across a lifetime, i.e., aging. A second aim of this paper is to probe the plausibility of such an approach by investigating whether face variants are gathering within distinct temporal clusters—similar to Mileva et al.,'s (2020) sub-prototypical representations. We will call such clusters*episodic prototype*s. The third aim of our paper is to investigate the process of learning faces and the corresponding prototype formation over a large time span of facial depictions.

## 2. Study 1

Study 1 was conducted to investigate whether the process of facial development across a large span of a human life is perceived as distinct *episodes*. We decided to use *unfamiliar* faces for Study 1 since the aim was to gain knowledge on episodes based on pure visual appearance that was not being confounded with semantic information about the respective persons (e.g., the depicted identity had a depressive episode for several years that could only be known for sure by a close friend or family member).

### 2.1. Method

#### 2.1.1. Participants

Twenty-four persons participated in the experiment (20 female; *M*  =  23.5 years, *SD*  =  5.0, range  =  19 to 37 years) on a voluntary basis. Most of the recruited participants were undergraduate students of the University of Bamberg and gained course credit to fulfill course requirements. All participants were naïve to the aim of the study and were not familiar with the faces presented. They were all assessed to be normal in terms of visual acuity, and color-vision tested via a standard Snellen Eye chart test and a self-made short version of the Ishihara color test, respectively. All participants gave written consent to participate in the study. All procedures and treatments of participants were in accordance with the Declaration of Helsinki. The study was in full accordance with the ethical guidelines of the University of Bamberg and was approved by the University Ethics Committee on 18 August 2017.

#### 2.1.2. Material

We collected 20 different facial presentations of four unfamiliar individuals (later called “models”) spanning a period of approximately 60 years on average (with approx. 3 years in-between different presentations). The first picture was always taken in the first year of the person's life; the last one was taken in the year 2015 in which we started this study, where the models were at an average age of 57.3 years with a *SD* of 1.5. This resulted in 80 facial presentations in total. Subsequently, each stimulus was graphically post-processed by converting it to grayscale and by blurring the contextual content (such as the background) using Adobe Photoshop CC 2021. Resulting images were finally scaled to 591 × 591 pixels. These images were mounted onto 24″ displays that showed two images side by side. The distance to the center of the pictures was 15 cm. A chin rest was used to align the line of sight with the center of the display (with the distance of the monitor to the eyes being approx. 60 cm). For each model we generated displays of all possible combinations of images per model, not taking the lateralization effects of the displays into account: leading to 2 out of 20 combinations  =  190 displays for each model, so 4 [models] × 190 [displays per model]  =  760 combined displays for the entire set of single stimuli.

#### 2.1.3. Procedure

Participants were asked to rate the *similarity* between both facial versions of one model on a 7-point Likert scale (ranging from 1  =  *very unsimilar* to 7  =  *very similar*). They were informed that the two presentations corresponded to the same person (without mentioning the variable age as the core difference between the presentations). Each trial started with a fixation cross (presented for 500 ms) in the center of the screen, followed by a blank screen (presented for 100 ms), followed by a display of two facial versions of one person which was present until the participant made a response. The entire procedure, including instruction and personal assessments, lasted approx. 40 min.

### 2.2. Results

For a better understanding of the analyses, we will first and foremost describe the methods used and the rationale for employing them. The main idea behind the face-space is that the unique exemplars of a face are encoded as points in this *n*-dimensional space and the distances between two points are analogous to the dissimilarities between the respective faces. The most typical face provides the centroid of a respective face-space, and less typical faces (or more distinctive faces) are located in a less densely clustered way in the periphery of that face-space. We suggested episodic prototypes corresponding to *temporal* clusters of facial representations. We followed this approach by employing an exploratory approach to generating a face space based on dissimilarity ratings for all exemplars. This was done by conducting cluster analyses. Research mainly focuses on two general types of cluster analyses: 1) Agglomerative hierarchical and 2) iterative partitioning clustering methods. Agglomerative hierarchical methods are similarity-based approaches (whereas dissimilarities are often called or operationalized by distances) wherein exemplars or cases start as individual clusters (number of clusters  =  number of cases); the most similar exemplars are then joined together step by step. This is achieved by the use of a predefined metric and a linkage criterion like *Ward's criterion* (Ward, 1963), which specifies the (dis)similarity of clusters as a function of the pairwise distances of observations in the cluster. This process is irreversible so that neither can joined clusters be changed nor exemplars be excluded anymore. In the case of iterative partitioning clustering (e.g., the *k*-means approach), the researcher has to determine the number of clusters before running the routine. This challenge can be solved by using several methods to find an appropriate number of clusters (see below for methods used in this study). Based on the pre-set *k* of clusters, the respective centroids are calculated, and exemplars are located to their nearest cluster centroid. This process continues until all the exemplars belong to those clusters to which they have the smallest distances to the respective cluster centroids. One advantage of this approach is that during the process of relocating the centroids, the cluster-membership of the exemplars can be exchanged. For the present study, we followed Milligan's suggestion (1980) of performing a two-step algorithmic routine: first, a hierarchical cluster analysis (Ward's method) to determine the number of clusters, followed by a partitioning clustering (*k*-means) for further optimization. All analyses were conducted by using the most recent R 4.0.4 (R Core Team, 2013) for *MacOS*, utilizing the *cluster* package (ver. 2.0.4) by Maechler et al. (2016). Additionally, besides the classical *elbow criterion* (a distinct drop of the within-groups sum of squares), we used the *gap statistic criterion* (Tibshirani et al., 2001) using the package *NbClust* (ver. 3.0) by Charrad et al. (2014) for an internal validation of the appropriate number of clusters. One main advantage of this criterion is that it can be applied to *any* clustering method.

**Figure 5.**
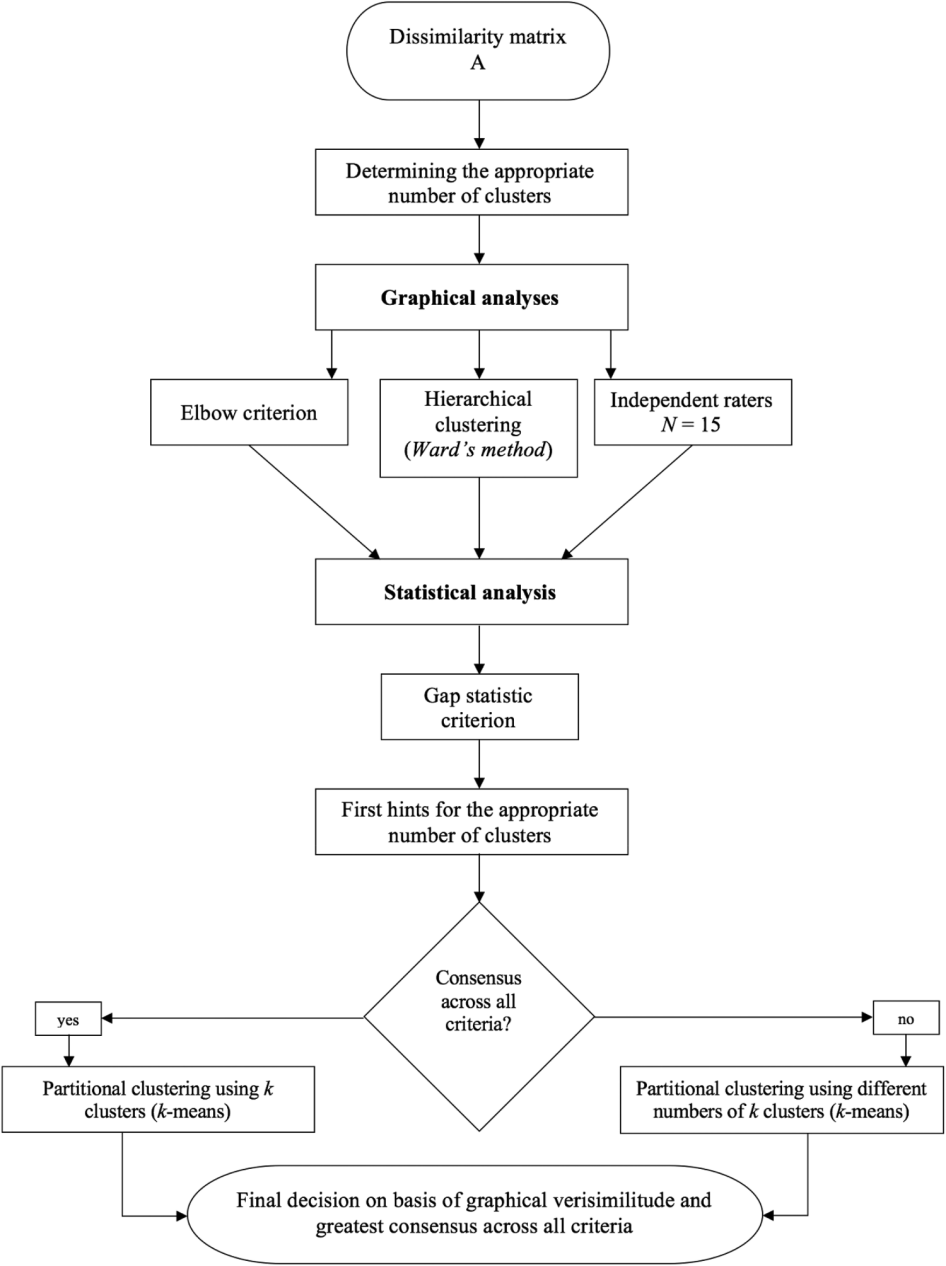
Flow chart demonstrating each step of our cluster analysis process.

For an external validation of the first step to automatically find the optimal number of clusters, we invited 15 independent raters (seven female, *M*  =  25.6 years, *SD*  =  2.2) to find clusters on the basis of the plotted face-space (without information about the exposure date). Note that outliers in face-spaces could easily arise from face-irrelevant aspects like differences in brightness, contrast, focus depth or background, hence they are processed and memorized as high distinctive faces (see e.g., Hancock et al., 2000). Most importantly, all these criteria should only be seen as a way of heuristically extracting cluster structures. Thus, we followed this general procedure in the following study of this paper. The whole process of analyzing the data in this way is shown in Figure 5.

Following former approaches toward generating two-dimensional face-spaces (e.g., Valentine, 1991) we chose *Euclidean distance* metrics. In the following, all results of the cluster analyses are presented per photographed person, shortly called “model” in the following.

#### 2.2.1. Model #1: Female, Born 1956 (Female1956)

**Figure 6.**
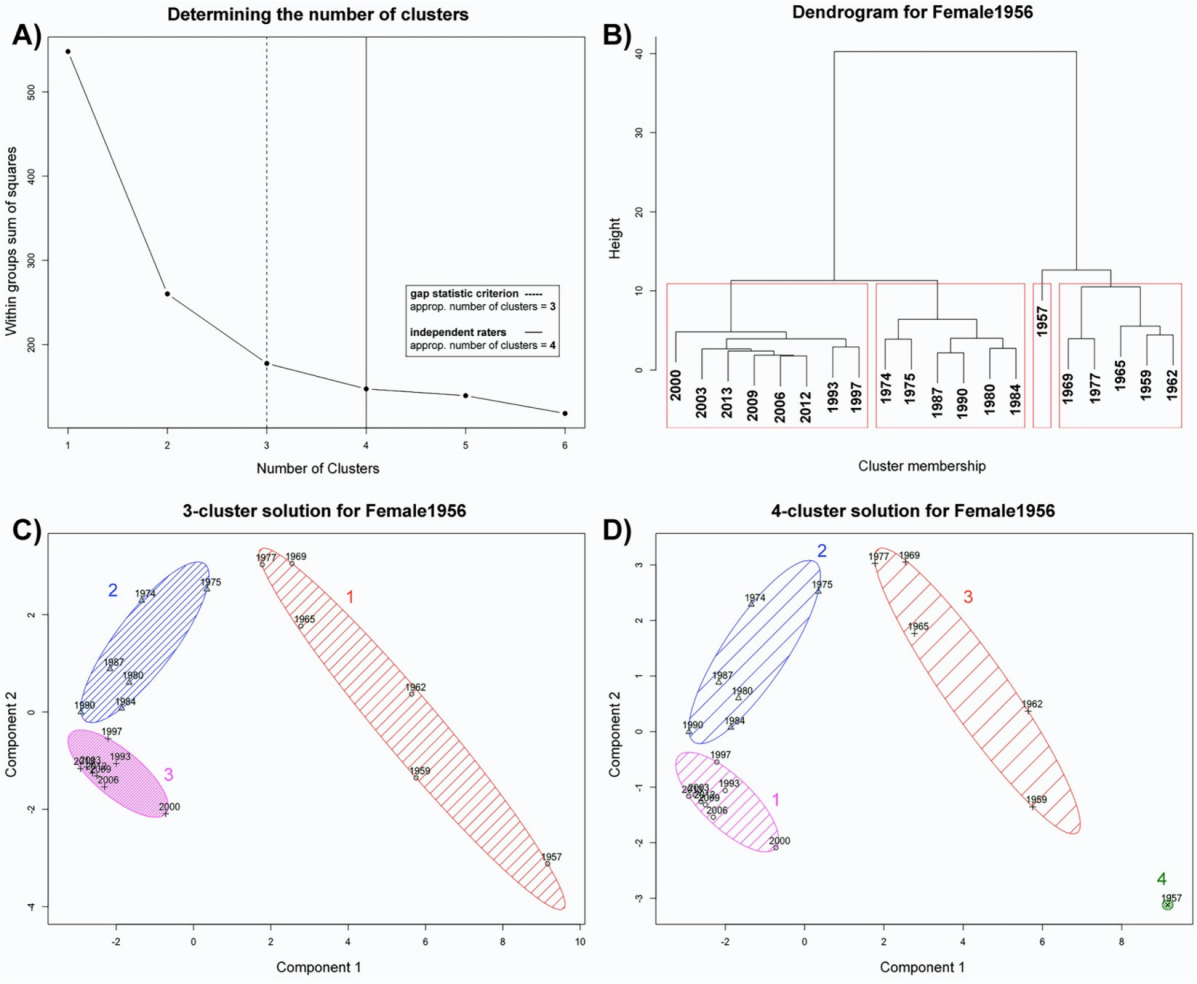
Model #1: *Female1956*. The gap statistic criterion (dashed line) revealed an appropriate number of three clusters (A), which deviates from the suggestion of the independent raters (solid line). B) shows the dendrogram (tree diagram) of a hierarchical cluster analysis (using Ward's method) with four clusters. C): Partitioning clustering (*k*-means) with three clusters reveals a clear sub-prototypical cluster pattern in the sense of an *episodic prototype*. In contrast, D) shows the preferred 4-cluster solution. Please note that one exemplar (year 1957: facial presentation of a baby from the first year of life) constituted its own cluster and was furthermore treated as an outlier.

As shown in Figure 6, analyses revealed clear sub-prototypical clusters in the outward facial appearance of the model (we will call this model *Female1956* in the following) providing first evidence that certain timespans reflect genuine prototypes. This stands in contrast to the idea of an *exhaustive prototype* spanning the entire life of a person. The elbow criterion, as well as the gap statistic criterion, revealed an optimal number of three clusters, whereas hierarchical cluster analysis (Ward's method) identified four clusters (see Figure 6)—this variety of optimal solutions was reflected by the human raters: most raters (*N* = 9) chose 4 clusters, five raters identified 3 clusters: Five raters and one single rater found a 5-cluster solution the most appealing. Most of the discrepancies might be put down to a potential separated cluster for just one exemplar which showed a non-frontally depicted baby in front of some rather face-irrelevant contextual information (see Figure 6, dendrogram and four-cluster solution). In fact, this exemplar and the resulting cluster can be treated as an outlier—this means we arrive at a 3-cluster solution (see Figure 6). We furthermore dropped the respective outlier face from consideration in Study 2.

#### 2.2.2. Model #2: Female, Born 1959 (“Female1959”)

**Figure 7.**
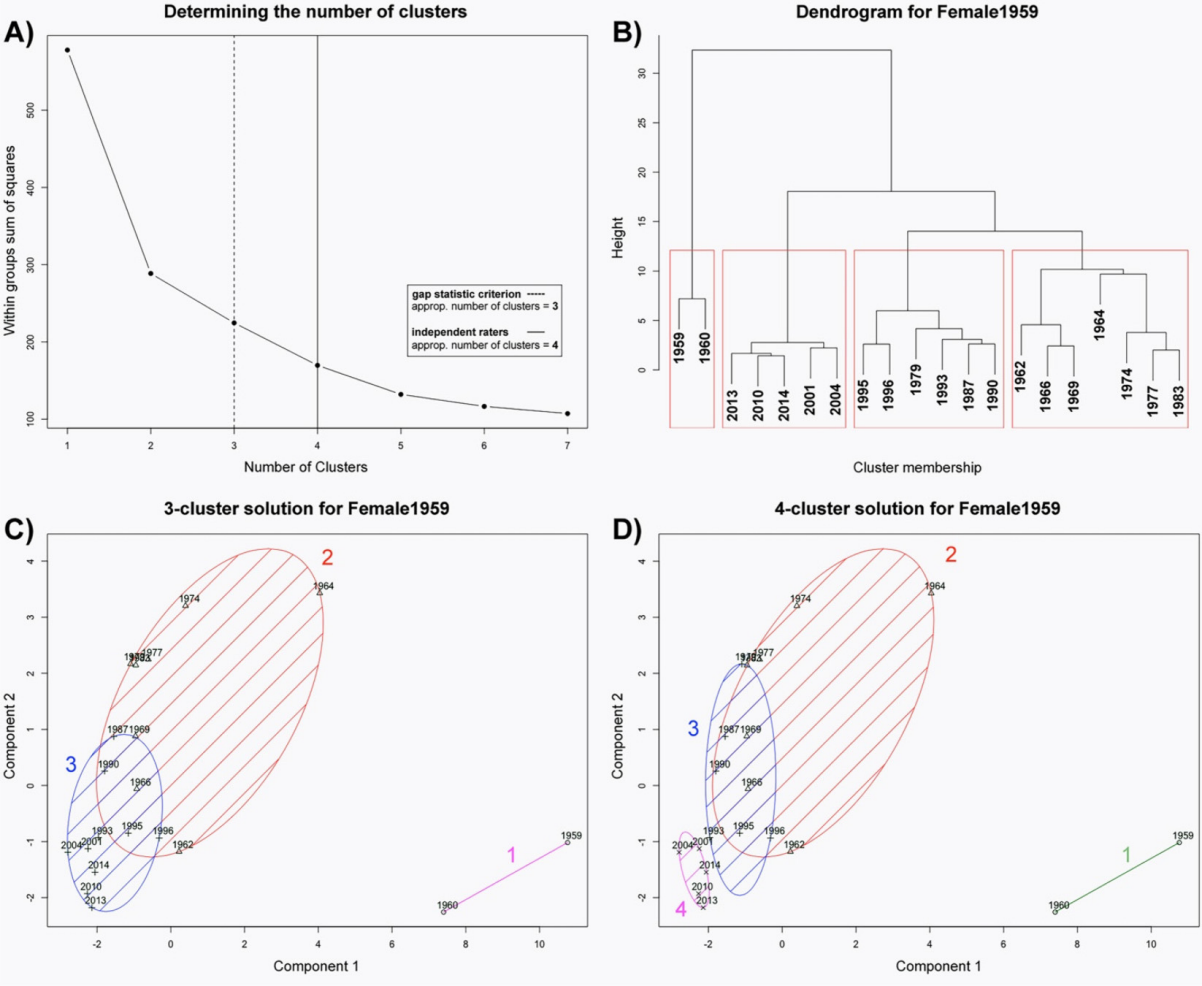
Model #2: *Female1959*. The gap statistic criterion (dashed line) revealed an appropriate number of three clusters (A) that deviates from the suggestion of the independent raters (solid line) as well as from the elbow criterion (there was still a significant drop in the within-groups sum of squares moving from a 3-cluster solution to a four-cluster solution). B) shows the dendrogram of a hierarchical cluster analysis (using Ward's method) with four clusters. As a result, we used the 4-cluster solution (D) in contrast to the 3-cluster solution (C) by conducting a partitioning clustering (*k*-means). This finding supports the idea of *multiple episodic prototypes* (there is a “baby cluster”, a “middle-age cluster” and a cluster for the most recent decade).

For the second female model (*Female1959*), we also found clear episodic clusters. Interestingly, as with the first female model, our results provide evidence for baby faces constituting their own class of facial representations: Presentations of the first two years were clustered separately (see Figure 7). However, as with the first female individual, the major challenge for the second female individual was to find an appropriate number of clusters. In this case, neither the *elbow criterion* nor the other criteria yielded a coherent solution. The *gap statistic criterion* suggested an optimal number of three clusters, whereas all independent raters suggested a number of four clusters. Relating to the *elbow criterion,* the 3-cluster solution was not necessarily an obvious solution (see Figure 7): there was still a significant drop in the within-groups sum of squares moving from a 3-clusters solution to a 4-cluster solution. Accordingly, on the basis of additionally considering the dendrogram and the hierarchical clustering, we decided to finally use a 4-cluster solution—with an additional fourth cluster for the latest episode (years 2001–2014; see Figure 7). All four revealed clusters represent a certain developmental episode: the first cluster could be identified as a “baby episode”, the second as a “youngster episode”, the third as a “middle-age episode” and the last cluster as an episode showing recent developments. Furthermore, we found a large gap between the first cluster (consisting of the years 1959 and 1960) and the second cluster, suggesting a qualitative development regarding outward facial appearance within the first two years.

#### 2.2.3. Model #3: Male, Born 1955 (Male1955)

**Figure 8.**
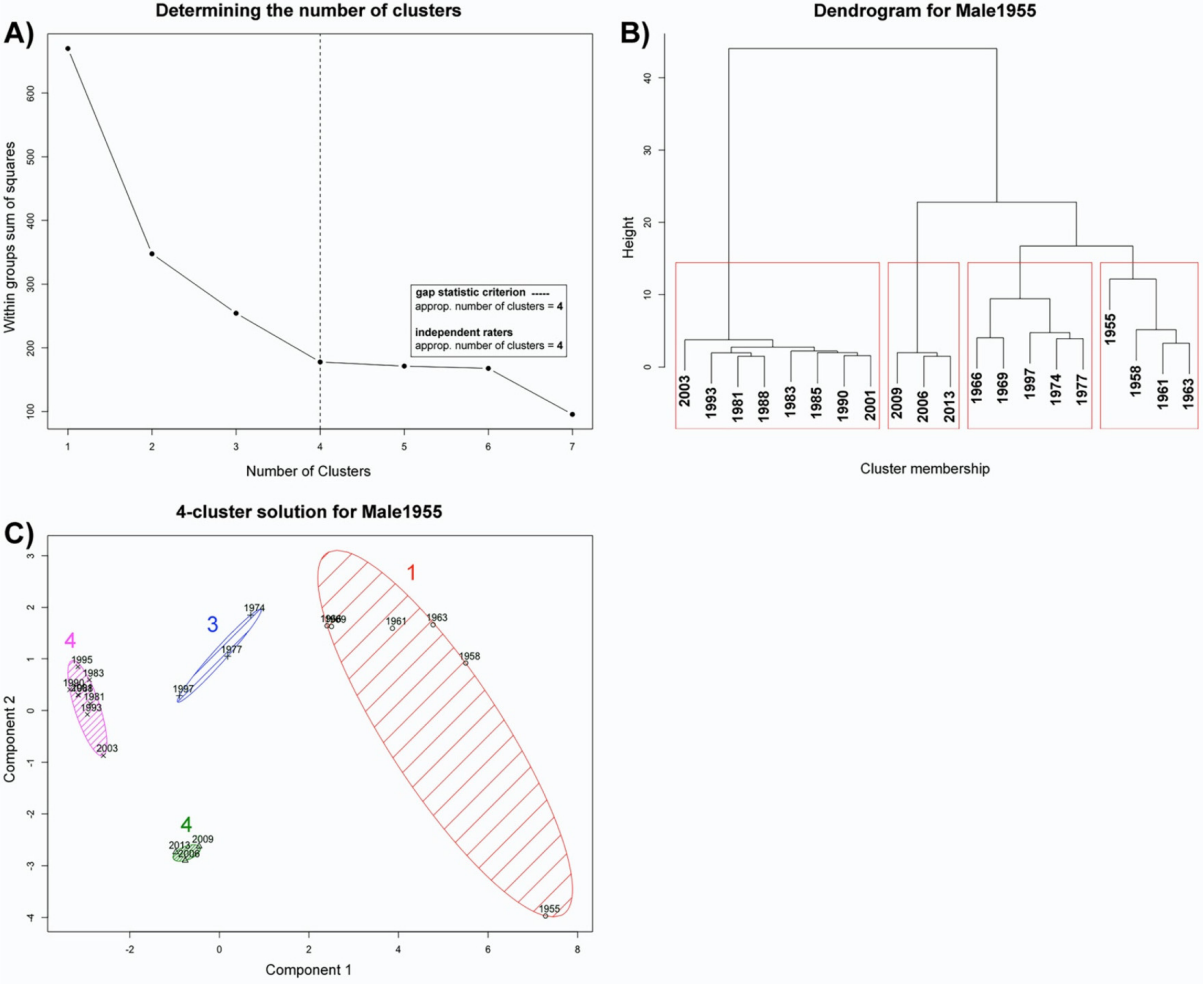
Model #3: *Male1955*. All used criteria (dashed line) revealed an appropriate number of four clusters (A). B) shows the dendrogram of a hierarchical cluster analysis (using Ward's method) with four clusters. C): 4-cluster solution by the conduction of a partitioning clustering (*k*-means), suggesting a clear chronological sequence in facial development over a timespan of around 60 years.

Figure 8 shows the results of the first male model (*Male1955*): In this case, we found a high consensus relating to the number of clusters. All criteria suggested a 4-cluster solution (see Figure 8). Accordingly, hierarchical clustering, as well as partitioning clustering, revealed a 4-cluster solution, again according to a “baby episode”, a “youngster episode”, a “middle-age episode” and an episode from the most recent decade (see Figure 8 for cluster memberships). Even if there was no single cluster for the baby face, the data for this individual showed a clear chronological sequence in facial development across the timespan, wherein almost every cluster reflects around a decade. Interestingly, the relatively large gap between the first cluster (∼1990s) and the third cluster (∼2000s) suggests a pronounced facial development in this model due to aging.

#### 2.2.4. Model #4: Male, Born 1957 (“Male1957”)

**Figure 9.**
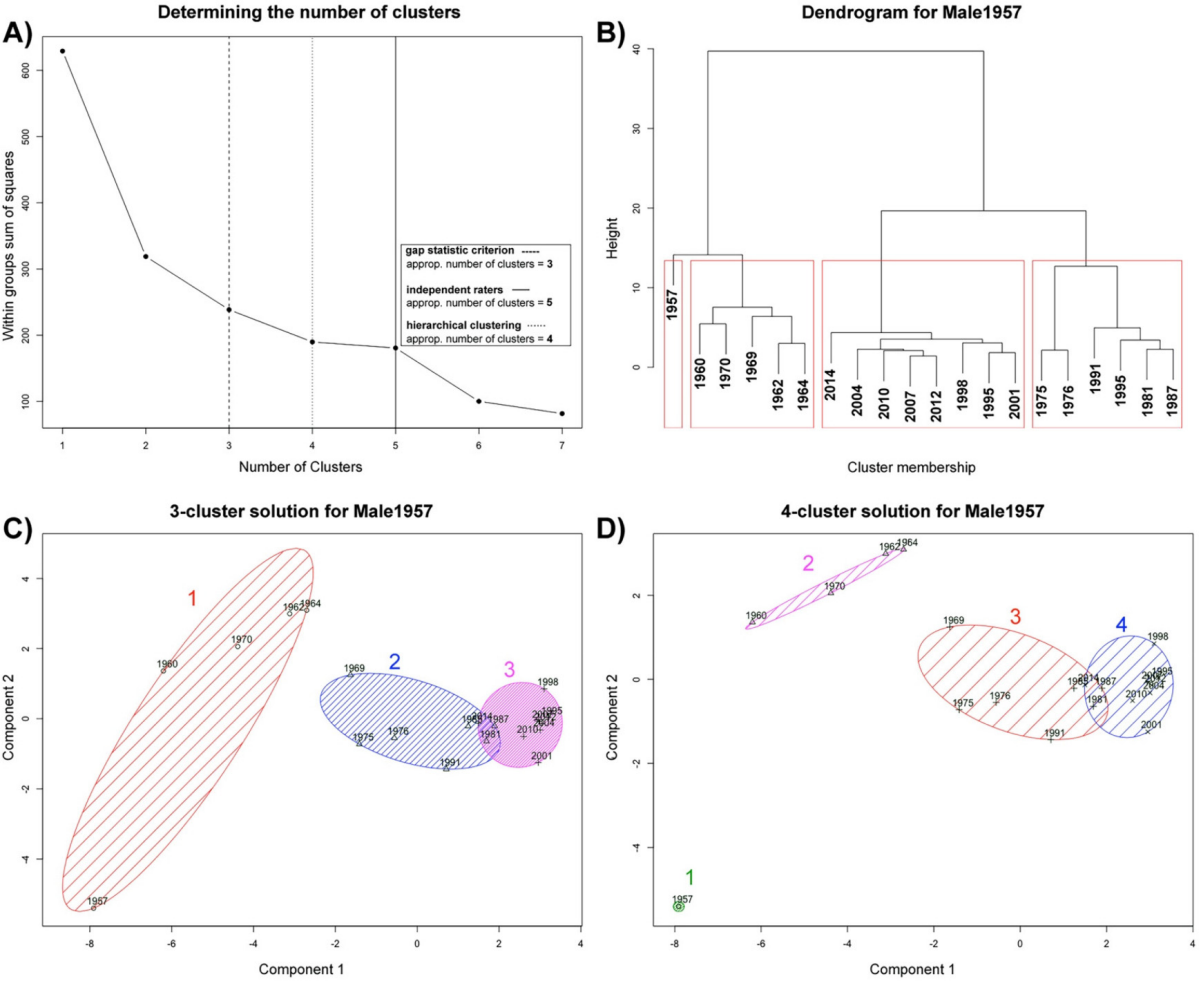
Model #4: Male1957: The gap statistic criterion (dashed line) revealed an appropriate number of three clusters (A) that deviates from the suggestion of the elbow criterion (there was still a significant drop in the within-groups sum of squares moving from a 3-cluster solution to a 4-cluster solution) as well as the result of the hierarchical cluster analysis (using ward's method) with four clusters (consider the dendrogram in B). As a result, we used the 4-cluster solution (D) in contrast to the 3-cluster solution (C) by conducting a partitioning clustering (*k*-means).

The mere visual inspection of the face space depiction for the second male model (“Male1957”) again suggests an episodic clustering pattern (see Figure 9). However, considering the criteria for determining the optimal number of clusters, the results were inconsistent (see upper quadrants of Figure 9). The *gap statistic criterion* suggested an appropriate number of three clusters, whereas the hierarchical clustering, as well as the elbow criterion, revealed a 4-cluster solution. Most raters were attracted to a 5-cluster solution (*N* = 8), followed by a 4-cluster solution (*N* = 4) and a 3-cluster solution (*N* = 3). As we did not observe a significant drop in the within-groups sum of squares from a 4- to a 5-cluster solution, we decided to conduct a partitioning clustering (*k*-means) with respect to a 4-cluster solution (see lower quadrants of Figure 9). As a result, the only difference between these cluster solutions was that the baby-face exemplar of the first year of the individual's life (“1957”) was treated as a single-cluster solution in the case of a 4-cluster solution. Reconsidering the dendrogram of the hierarchical clustering, we decided to use a four-cluster solution, because the baby-face exemplar “1957” had the highest dissimilarity to all other exemplars and would have led to an extreme within-cluster variance of the first cluster (see Figure 9). As described before, we found clear temporal clusters reflecting the individual's facial development across the timespan of his life. The results also suggest that baby faces constitute their own facial category, especially from the first year.

### 2.3. Discussion

The main aim of Study 1 was to investigate the plausibility of episodic prototypes. Supporting the initial evidence of recent research (Mileva et al., 2020), we indeed found that faces are *episodically clustered* and that these clusters reflect a typical temporal facial development of a person. This finding emphasizes the weaknesses of rather exhaustive-based prototypes models to describe how faces might be mentally organized. Our results suggest that facial development across about 60 years is optimally divided into approximately four clustered episodes, whereas baby faces constitute their own episode of facial representations next to a “youngster episode”, a “middle-age episode” and an episode from the *most recent* decade. In the given cases of people being about 60 years old, this latter episode coincides with an “elderly episode”. It is quite probable that extending the age range, even more, will lead to more clusters, for instance, “old” and “very old” episodes. Except in the case of the model Male1955, we found that such episodes cover different periods: the baby episode covers just the first ten years, whereas the third prototype constituted presentations of recent times. It seems reasonable to further assume that personal experience and the frequency of encountering a person plays a role in the extension, level of detail and quality of such prototypes. More in detail, frequent personal contact with a person leads to finer graded representations: e.g., even during the first three years of development, parents typically have the impression that their baby changes its outward appearance quite quickly, so the updating of an existing episodic prototype or the formation of a new one seems to be needed more frequently than for persons who encounter the baby only occasionally.

Study 1 revealed reasonable temporal clusters that rely on visual similarity, suggesting that the episodic clusters are more suitable to describe the facial representation of a certain person in the sense of an *episodic facial prototype*. Following recognized scientific theories, prototypes are defined as results of principal components or averages of given exemplars spanning a face-space that corresponds to one's facial representation of a certain person (see e.g., Benson and Perrett, 1993; Burton et al., 2005; Busey, 1998; Gao and Wilson, 2014; Reinitz et al., 1992; Webster et al., 2004; Webster and MacLeod, 2011). However, these models neglect temporal facial developments; hence the respective prototype is highly dependent on *temporal* aspects like the process of aging. Considering the results of Study 1, we propose the concept of *multiple prototypes*—or, more specifically, the better economy of *episodic prototypes* per identity.

## 3. Study 2

Based on the results of Study 1, we investigated further how faces could be mentally organized in episodic clusters in terms of *episodic prototypes*. We postulate that the most recent facial representation of a given person corresponds to a sum of more or less “recent*”* encounters with the respective face—in other words: the best representation of a living person might be the most recent episodic prototype. This idea is inspired by general memory theories and is further validated by, e.g., facial aftereffects (see e.g., Carbon and Ditye, 2011, 2012). We tested this idea by employing participants who had experienced a long-term history of learning so-called “personally familiar faces” (Carbon, 2008), in the of really deep visual knowledge employing social interaction. To realize this, we invited first- and second-degree relatives of the respective models who provided the stimulus material of Study 1 and allowed them to rate the *prototypicality* of all episodic prototypes. The rationale behind this approach is that if we take participants who are highly familiar with the provided face material due to the fact of knowing the depicted individuals for a long time, we expect that the prototypicality will increase the closer a facial depiction is to the current image a person shows. In other words, the best representation of a living person might be the most recent episodic prototype.

### 3.1. Method

#### 3.1.1. Participants

We invited thirty-eight first- and second-degree relatives of the models from Study 1 (approx. *M*  =  9.5 relatives per individual), 17 female; *M*  =  54.9 years, *SD*  =  13.1, range 24 to 74 years. All participants had known the respective model for at least 20 years and were in regular contact with them (please note that this combined criterion is essential for Study 2; hence the facial prototype is highly dependent on temporal aspects like *how long* we have known and *how often* we have experienced the respective person).

#### 3.1.2. Materials

Based on the data from Study 1, we generated *episodic prototypes* by morphing respective faces comprising these prototype clusters via Abrosoft^©^ FantaMorph V5 (5.4.6). We defined a set of 63 facial landmarks which are unambiguously identifiable and which have proven successful across a wide range of studies at our lab for more than a decade. The entire material consisted of all unique exemplars of Study 1, plus the respective *episodic prototypes* (the morphed representatives of the extracted clusters) and the *exhaustive prototype* (the morphed representative of all exemplars of a model). On the one hand, cluster analyses of Study 1 revealed that presentations of babies constituted separate clusters consisting of single facial presentations and large distances to other presentations that may support our hypothesis of episodic prototypes representing each EoL. However, on the other hand, we deliberately decided to exclude this baby cluster to avoid artificial cluster caking. Consequently, we yielded 3 *episodic prototypes* (*youngster prototype*, *middle-age prototype*, *recent prototype*)  +  1 *exhaustive prototype*  +  20 individual exemplars  =  24 facial exemplars per model set.

#### 3.1.3. Procedure

Each participant was assigned to the set of the respective familial model. The participant was asked to rate the typicality (as an operationalization of prototypicality) of each exemplar on a 7-point Likert scale (ranging from 1 = *very untypical* to 7 = *very typical*). We provided the participants with an explicit example: “You have now learned the face of a person. Please think of this person. You might have a picture of this person in your mind. In this case, please match the following presentations with your picture in your mind. “very typical” means that the presented picture fits “very well” to your mental representation of the person. “very untypical” means that the presented picture fits “very poorly” to your mental representation of this person”. The order of trials was randomized for each participant. The whole procedure lasted approx. 15 min.

### 3.2. Results

**Figure 10.**
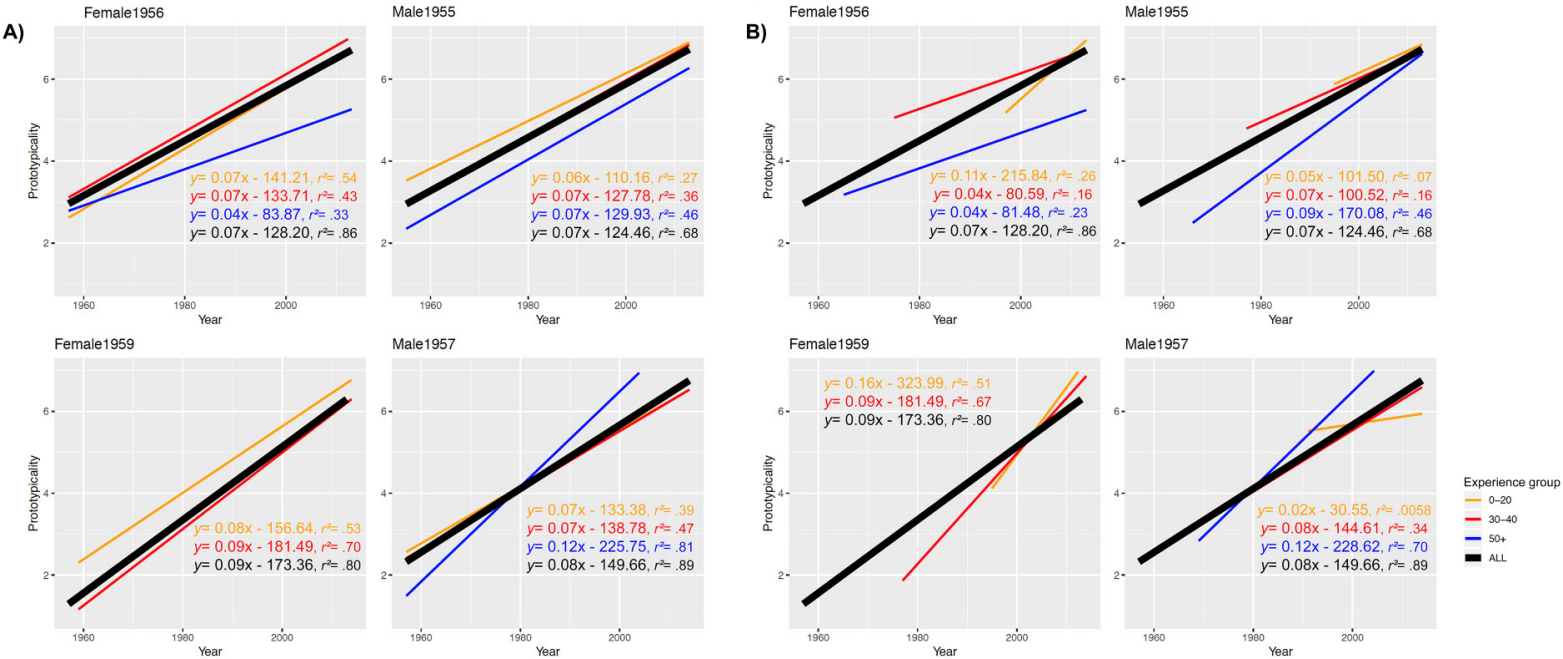
A) regression analyses with the year of image as independent and prototypicality as the dependent variable for each model, split by “experience group” (how many years the participants were personally familiar with the respective model) per employed. Additionally, we show the linear regressions for taking all experience groups together (“ALL”—bold black lines). Regression equations are based on averaged data across all participants with respect to the experience group demonstrating a clear linear relationship. Further details on the statistics (*r^2^* values were calculated on the basis of the averaged Fisher's *Z* values of the individual regressions per participant) of the regression analyses can be found in Table 1. B) Regression analyses with the year of image as independent and prototypicality as the dependent variable for each model, split by “experience group” (how many years the participants were personally familiar with the respective model) per employed model plus an overall age-group model (“ALL”). Regression equations are based on averaged data across all participants concerning the experience group showing a similar linear pattern but decreasing with the level of experience with a face (“0-20” < “30-40” < “ + 50” years of experience).

In a first step, we investigated the hypothesis that we have multiple episodic prototypes of a certain individual, and that more recent prototypes are stronger mentally represented and hence should be rated as more typical compared to older presentations. Accordingly, we tested whether the *age* of the unique exemplars could predict the perceived prototypicality. Four separated regression analyses (one per model) revealed that *age* satisfactorily predicted the perceived *prototypicality,* see Figures 10a and b as well as Table 1—we were able to reveal a close relationship between both variables when we rigidly used age as predictor as well as when we specifically fed in only the respective age spans which were reflected by the duration of familiarity of the different experience (three) groups (i.e., being familiar with the person for 50 + years, 30–40 years or less than 20 years). Although this finding might not be ultimately surprising at first, the robust data pattern provides strong indications of the episodic quality of facial representations and prototypes.

**Table 1.** Relationship between the perceived *prototypicality* and the respective *year* of the respective depiction across all models. Calculated *R^2^* values are based on the averaged Fisher's *Z* values of the individual regressions per participant. Data was split into three age groups (“Experience Group”) indicating the duration of personal experience with the relative (model): 0−20 years, 30−40 years and 50+ years plus an extra section for data on all experience groups. *R*^2^ indicate correlations for time series with different experience levels: whether participants’ ratings are based on all data (“all years”) or actual personal experience with the respective person / model (“experienced years”). In the case of *Female1959* there was no data available (n/a) for 50 + years, as for this individual there were no relatives with experience of that timespan

	Experience Group							
	0-20 years		30-40 years		50 + years		all experience groups	
Model	all years	experienced years	all years	experienced years	all years	experienced years	all years	experienced years
*Female1956*	.63	.36	.52	.13	.44	.34	.54	.21
*Female1959*	.54	.51	.74	.73	n/a	n/a	.68	.66
*Male1955*	.36	.08	.45	.18	.53	.59	.45	.26
*Male1957*	.06	.06	.64	.32	.81	.70	.60	.34

In view of the fact that, for instance, younger relatives (experience group 0–20 years) could only experience the respective model for between a couple and up to 20 years, we further only considered data from years of actual experience (see Figure 10b)—this was done to test the previous analyses of potential artifacts. However, we revealed a very similar pattern: recent exemplars were perceived as more prototypical, whereas older exemplars were perceived as less prototypical—again, reasonable and consistent linear fits with positive slopes (see Table 1).

**Figure 11.**
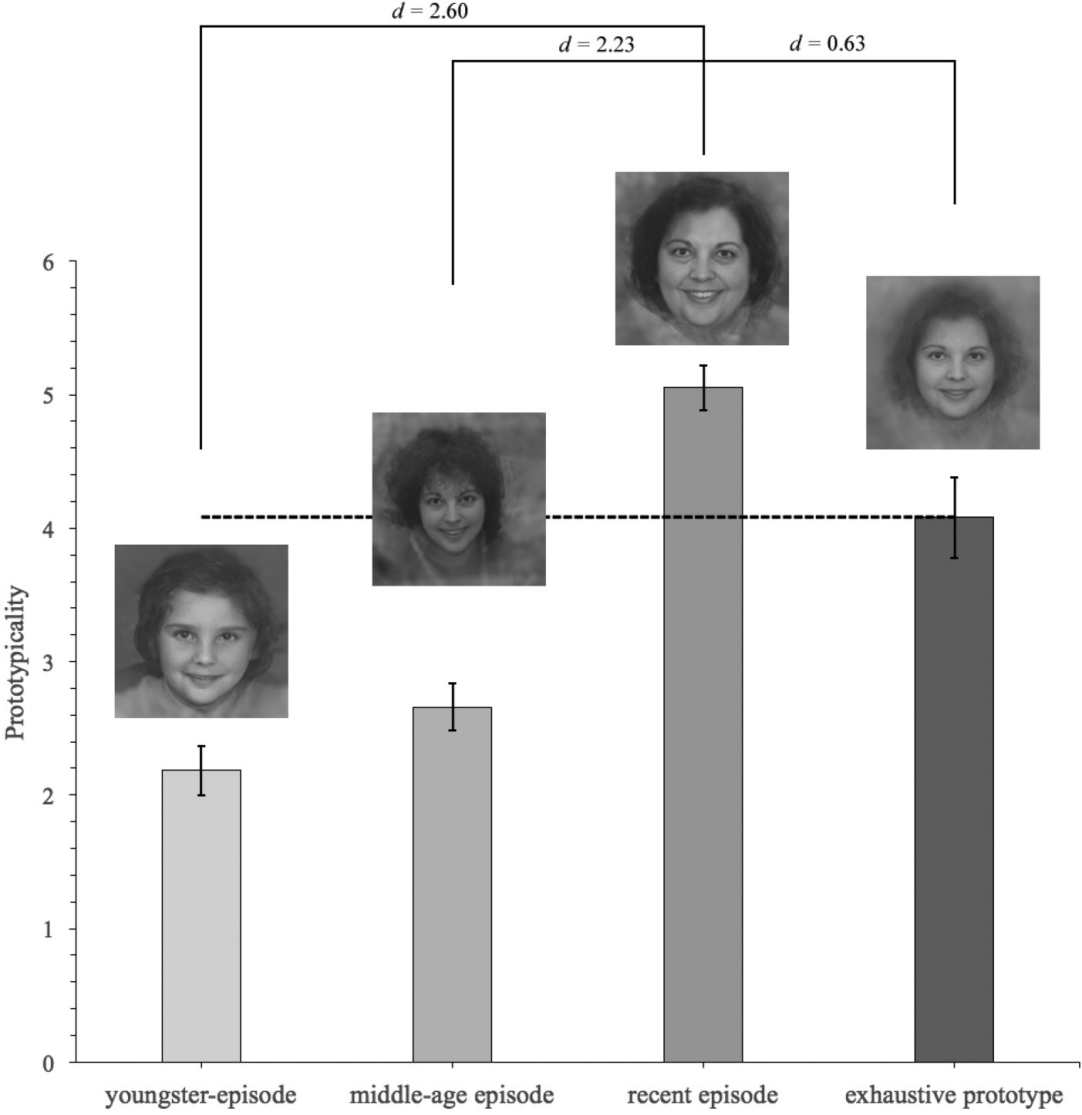
Demonstration of prototypicality ratings for *episodic* and *exhaustive* prototypes (representations are only examples, here *Female1959*). Observers rated morphs of actual decades as significantly more prototypical. Error bars indicate  ±  1 standard error from the mean. Dashed line indicates the mean level (prototypicality) of the *exhaustive prototype*.

Relating to the *episodic prototypes*, we postulate that prototypes from recent decades are mentally activated to a higher degree than prototypes which are rarely referred to. Accordingly, we investigated whether facial representations are temporally weighted as well as whether *prototypicality* was dependent on the model's age by conducting a two-factorial repeated-measures analysis of covariance (rmANCOVA) with the within-subject factor *episodic prototype* [*youngster prototype*, *middle-age prototype, recent prototype, exhaustive prototype*] (note that for this analysis, prototypicality ratings were averaged across the four models)*,* the between-subject factor *participants’ sex* to investigate whether female vs. male relatives share the same prototype*,* and the covariate *participants’ age* to investigate the impact of the amount of personal experience with the respective prototype. A univariate approach with Huynh-Feldt correction (Huynh & Feldt, 1976) for the degrees of freedom (*df*) was used (correction factor ε), which should be applied if ε is >.75 (Girden, 1992), but for a better flow of reading, the original value of the *df* is reported. Partial η^2^ (η_p_^2^) is reported as a measure of association strength. An *α*-level of .05 was used for all analyses reported in this paper and all reported *p*-values are two-tailed. Pairwise comparisons (two-tailed two-sample *t*-tests) and respective Cohen's *d* were additionally calculated (see Figure 11). Further analyses were conducted with a focus on the simple main effects. All assumptions for conducting an rmANCOVA were sufficiently fulfilled: independence of observation, normality of distribution of residuals, linearity of regression (linear relationship between the dependent variable and the independent variable), homogeneity of regression slopes as well as the homoscedasticity across and within all groups. All analyses were conducted on the R 4.0.4 (R Core Team, 2013) platform for macOS.

Analyses revealed that *episodic prototypes* had a significant effect on the perception of *prototypicality, F* (3, 105)  =  4.86, *p*  =  .006, η_p_^2^  =  .12, ε  =  .96, where the *episodic prototype* of *recent decades* (*M_recent_*  *=*  5.05, *SD_recent_*  *=*  1.04) yielded the highest *prototypicality* ratings, followed by the *exhaustive prototype* (*M_exhaustive_*  *=*  4.08, *SD_recent_*  *=*  1.91, *d*  *=*  0.63), the *middle-age prototype* (*M_middle−age_*  *=*  2.66, *SD_middle−age_*  *=*  1.10, *d*  *=*  2.23) and the *youngster prototype* (*M_youngster_*  *=*  2.18, *SD_youngster_*  *=*  1.60, *d*  *=*  2.60). This suggests that facial prototypes are highly temporal-dependent and that these representations are weighted toward recent experiences with the respective face. More importantly, it clearly contradicts the thesis that artificial *exhaustive prototypes* actually correspond to one's facial representation (see Figure 11). We further found a small but significant main effect of the between-subject variable *participants’ sex*, *F*(1, 35)  =  4.47, *p*  =  .042, η_p_^2^  =  .11 with higher prototypicality ratings for female observers*.* However, there was no interaction between the *participant's sex* and the *episodic prototypes* indicating that male and female observers shared the same prototype, *F*(3, 105)  =  1.31, *p*  =  .275, *n.s*. With respect to the covariate *participants’ age* we found no significant effect*, F*(1, 35) < 1, *p*  =  .386, *n.s.*, suggesting an own-age-independent genesis of prototypes (in the sense that younger and older relatives shared the same facial prototype, respectively).

### 3.3. Discussion

**Figure 12.**
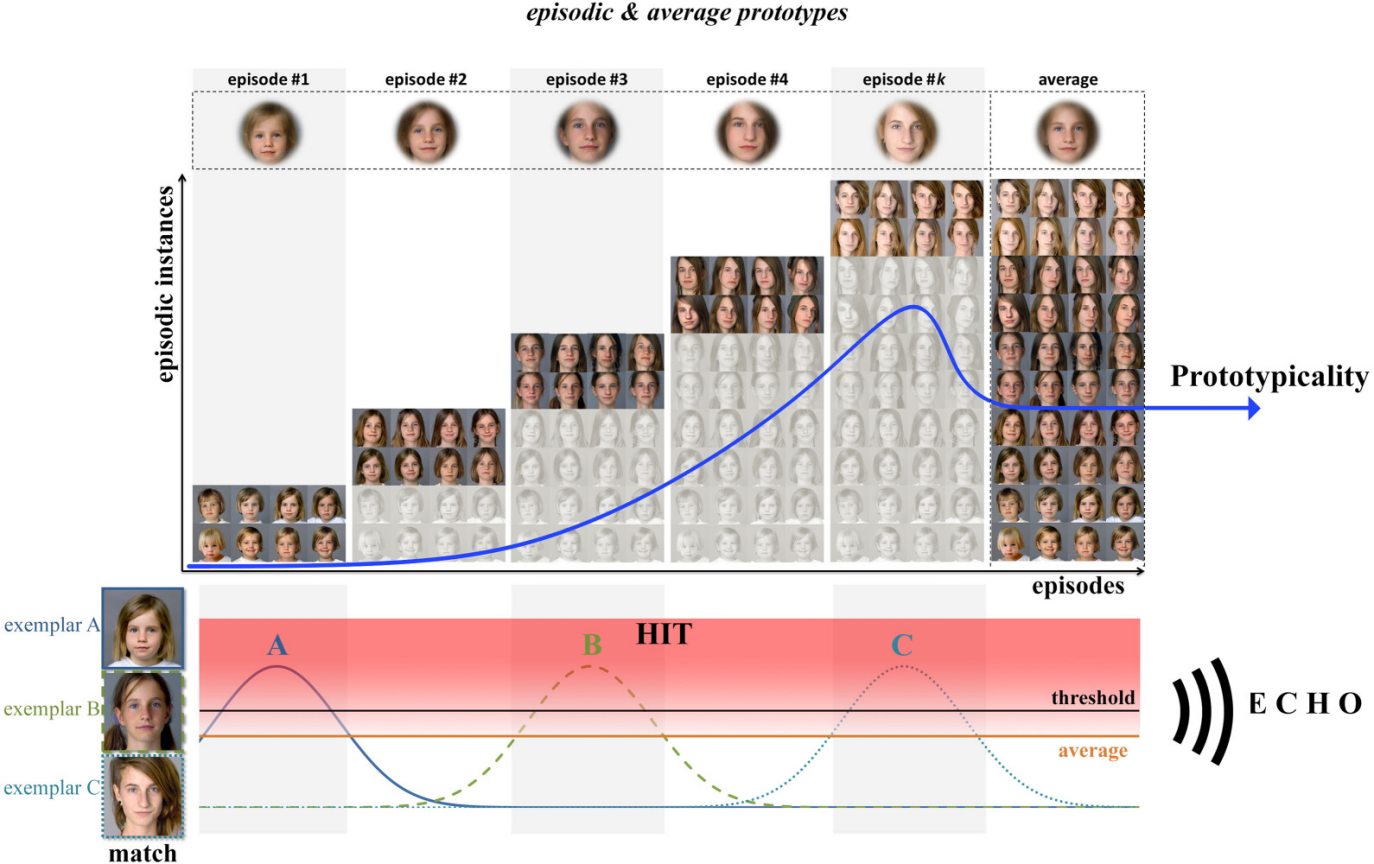
The *Episodic Prototypes Model (EPM).* With respect to *exemplar-based representation models* (e.g., the *MINERVA 2 model*), *episodic prototypes* represent outward facial appearance during certain episodes of human life (EoL). Such episodes refer to distinctive sub-prototypical representations. Given a new exemplar (A, B or C) the *similarities* to all exemplars stored in the storage are calculated in a first step. A so-called “*echo”* is calculated by adding all the weighted features of inherent exemplars relating to a representation. The new object is then matched to the representation or prototype with the strongest echo (red “HIT”-area: a hit is equivalent to a successful match). This matching procedure is much more efficient and valid as it is based on *episodic prototypes*. In contrast, the *average prototype* (orange line and right column) only produces a mid-level echo and does not engage a distinct match (below a pre-definite threshold; black line). For example, *exemplar A* is matched to the *first* episode (first *episodic prototype*, first column); hence the sum of all exemplars/instances of this episode produces the most extensive echo to *Exemplar A*. Note that there is a decrease and an increase in the echo moving from one episode to the next (in the case of *Exemplar A*, symbolized by the blue graph), respectively. The increasing echo coincides with the *average's* echo at the edge of an episode. The echo increases exponentially to such an extent that there is some echo in the following episode. However, this echo falls far below the echo of the *average prototype* until it disappears completely. This routine means that one person is potentially represented by multiple episodic prototypes which reflect one specific EoL. In contrast to the *exhaustive prototype*, episodic prototypes of recent EoL are perceived as most prototypical, suggesting an adaptation towards recent experiences with the respective face (blue arrow). For this illustration, we used *k* episodes; however, please note that the number of episodes certainly depends on many factors (e.g., the quality and quantity of events where we were able to become familiarized with the target person's face).

In Study 2, we investigated whether the clusters of Study 1 can be taken to describe one's facial representation in the sense of an *episodic facial prototype.* The main challenge in addressing this question is that it can only be answered validly by an already existing face-space (or an already existing facial representation). There are usually two ways to generate a facial representation: 1) Familiarization by an experimentally controlled learning task (similar to Murphy et al., 2015; White et al., 2014) or 2) familiarization by a lifelong learning-/experience-based process (e.g., in the case of relatives). Using an approach based on lifelong learning/experiences has the advantage that it accesses the ecological variations of an individual's face (in the sense of an individualized idiosyncratic face-space). This is in contrast to face-spaces spanning different persons and different dimensions based on (dis)similarity ratings alone (e.g., Busey, 1998; Johnston et al., 1997). However, besides the fact that recent studies which followed more sophisticated approaches by considering individualized, idiosyncratic factors (e.g., Burton et al., 2016; Kramer et al., 2017; Murphy et al., 2015; Ritchie and Burton, 2017; Young and Burton, 2017; but see Mileva et al., 2020) did *not* consider or control *temporal* aspects, the second approach reflects a more lifelike process of learning faces (e.g., relatives, very close friends). Accordingly, the results of Study 2 strongly suggest that facial representations are closely dependent on *temporal* aspects in which *recent* experiences with the respective face were significantly rated as most prototypical (*recent episodic prototype*). One mechanism for this could be the higher probability of the most recent episodic prototype being activated as it reflects the latest or mist updated information for present and future recognition requirements: an alternative mechanism could be an adaptation towards recent experiences (see e.g., Carbon and Ditye, 2011). Interestingly, the revealed pattern for younger relatives who in fact could have experienced the respective face for only a few years was very similar to that of older relatives having had much more experience across decades. This points to a universal mechanism where the episodic prototype containing more recent outward appearances, actually those which are most important for recognizing people in everyday life contexts, is primarily activated and referred to when we think of the prototypical outward appearance of a target person. In order to be constantly “up to date,” we have to assume that prototypes are dynamically generated using a rapid update mechanism. In most real-world contexts, especially when we think of the natural familiarization of people we know personally, such prototypes are chronologically ordered, and this order does not just reflect similarity issues (see Study 1); and so facial prototypes and respective representations will rely on *temporal* aspects. Such *episodic prototypes* can most accurately and economically represent certain phases in the development of a face, particularly the most recent episode in a face's life. As shown by Study 2, the most recent episodic prototype will even outperform an *exhaustive prototype* which had been repeatedly demonstrated to be already quite powerful in representing faces (see e.g., Burton et al., 2005; Gao and Wilson, 2014; Jenkins and Burton, 2008). Accordingly, we postulate the *Episodic Prototype Model (EPM*) to describe the natural formation of facial prototypes and representation of faces with respect to strong visual changes induced by temporal aspects (e.g., aging, but see general discussion for further influential factors), see Figure 12.

The *EPM* (see Figure 12) combines the strength of *exemplar-based representation models* (e.g., the *MINERVA 2 model*) and the idea of different densities in the face-space induced by certain prototypes which are defined by EoL. Such episodes refer to distinctive sub-prototypical representations. Given a new exemplar, the *similarity* to all exemplars stored in memory is calculated in a first step. A so-called “*echo”* is calculated by adding up all weighted features of inherent exemplars relating to a representation. The new object is then matched to the representation or prototype with the strongest echo. This matching procedure is much more efficient and valid as it postulates *episodic prototypes*. In contrast, the *average prototype* only produces a mid-level echo and does not engage a distinct match. For example, a baby face is matched to the *first* episode of human life (“baby episode”); the sum of all exemplars/instances of this episode produces the most extensive echo for this new face. Note that there is a decrease and an increase in the echo moving from one episode to the next (see Figure 12), respectively. The increasing echo coincides with the *average's* echo at the edge of an episode. The echo increases exponentially so that there is some echo in the following episode. However, this echo falls far below the echo of the *average prototype* until it disappears completely. This routine means that one person is potentially represented by multiple episodic prototypes that reflect one specific EoL. Although the *EPM* relies on a rather *exemplar-based* approach, it could be understood as a *hybrid model* consisting of *norm/average*—as well as *exemplar-based* aspects. Firstly, a prototype is reflected by a sum or an abstraction of single exemplars that reflect a *specific* timespan in a human life (*episodic prototypes*) what is in line with *partial* abstraction approaches such as the varying abstraction model (Vanpaemel & Storms, 2008). But secondly, in accordance with rather *exemplar-based models*, it postulates that there is *no* certain or exhaustive prototype which reflects a respective person's face. Instead, the model suggests that a person's face is represented by *multiple episodic prototypes*.

With respect to the first two studies, it is important to note that learning facial variability across longer time spans is very much dependent on the beholder's experience. In Study 1, participants were (pre-experimentally) unfamiliar with the presented faces, and the resulting episodic prototypes are therefore *not* based on personal experiences. From this point of view, one could argue that the pattern of clustering in Study 1 could be different from personally experienced faces since episodic prototypes of relatives may rely on different factors. Accordingly, the resulting episodic prototypes in Study 1 may not reflect the actual facial representation of the relatives. Interestingly, concerning exemplar-based analyses (see regression analyses in Figure 10a and 10b), Study 2 revealed that even across different levels of experience with a respective face (e.g., 0–20 years vs. 50 + years) the pattern remains quite similar: depictions of younger periods were perceived as less prototypical whereas recent presentations were perceived as more prototypical. However, the question remains whether and how personal facial experience affects the genesis of episodic prototypes. Accordingly, to answer this question, we conducted a third study with a focus on the genesis of episodic prototypes of faces that are personally experienced.

## 4. Study 3

In contrast to the first two studies, we actually let participants learn unfamiliar faces to investigate the process of prototype formation within a controlled lab experiment. For clarification of our idea, we would like to provide an example: imagine that you had a school friend, and you only knew him from his 10^th^ to 20^th^ year of life. Thirty years later (he is now around 50 years old), you meet him somewhere by accident. It is highly probable that you will have some difficulties in recognizing this person since you have a high level of experience with his younger face but no recent experiences with his current facial appearance. Accordingly, it may be suggested that your facial representation would refer to a rather young-looking person and, consequently, his recent facial appearance would be rated as less prototypical since he will have changed dramatically across the years (e.g., facial developments such as skin aging, wearing a beard, grey hair, partially tattooed and pierced, probably also showing more facial mass). In contrast, another friend who became acquainted with him for the first time only ten years ago would only have a limited, decade-long perceptual knowledge of him. Logically, in his case, the facial representation is solely based on recent experiences (ideally given that even older photos are not known).

Accordingly, Study 3 was conducted to investigate whether persons with different levels of experience with a given face have different or similar episodic prototypes. Following the idea of the *EPM* that facial prototypes are updated very quickly, we further investigated the perceived prototypicality of actually experienced faces vs. faces which have not been experienced before (e.g., you only know a person from teenage times, but now you meet this person after 30 years).

### 4.1. Method

#### 4.1.1. Participants

Twenty-four persons participated in Study 3 (15 female; *M*  =  23.2 years, *SD*  =  3.4, range 18 to 33 years) on a voluntary basis. They were undergraduate students of the University of Bamberg and gained course credit to fulfill course requirements. All participants were naïve to the aim of the study and were not familiar with the presented faces. They were all assessed as being normal in terms of visual acuity, and color-vision tested via a standard Snellen Eye chart test and a self-made short version of the Ishihara color test, respectively. All participants gave written consent to participate in the study. All procedures and treatments of participants were in accordance with the Declaration of Helsinki. The study was in full accordance with the ethical guidelines of the University of Bamberg and was approved by the University Ethics Committee on 18 August 2017.

#### 4.1.2. Materials

**Figure 13.**
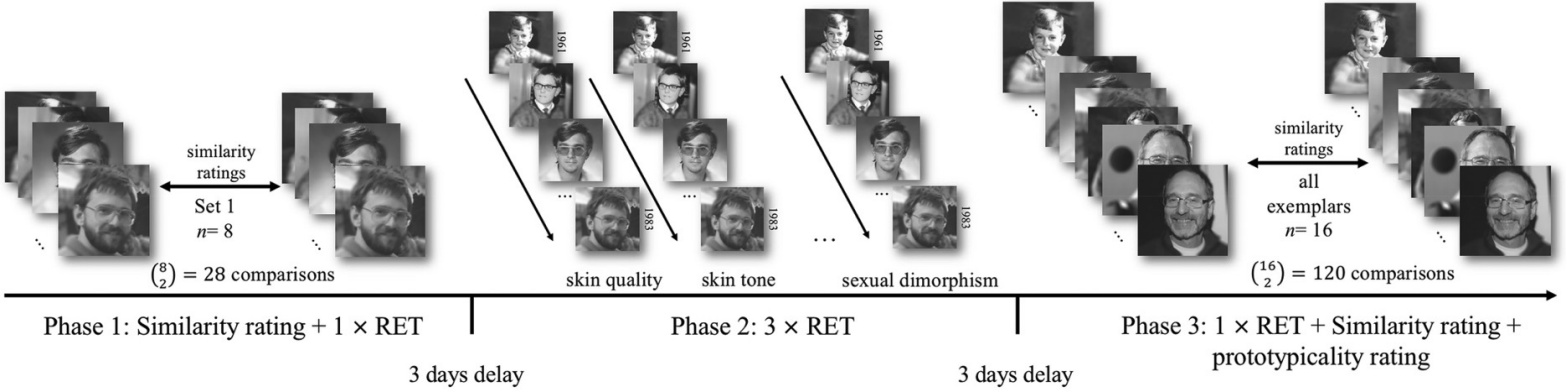
Design of Study 3. Participants were randomly assigned to one model (here demonstrated with model *Male1955* and Set 1). In Phase 1, they had to rate the similarity between all depictions within the respective set (in this example Set 1) plus learn the faces via RET. In Phase 2, the RET was repeated three times. Phase 3 started with one RET session followed by similarity ratings across all exemplars (Set 1 plus Set 2). Afterward, they had to rate the prototypicality across all exemplars plus *episodic prototypes* plus *exhaustive prototypes*.

We used the same material from Study 1 and selected 16 different facial presentations of each model. This resulted in 64 facial presentations in total. Subsequently, for each model we created two different exposure conditions (Set 1: Eight younger presentations vs. Set 2: Eight recent presentations, see Figure 13). Because baby faces seem to be represented differently, the selected presentations started from the age of five years with approx. Three years in between different presentations). The first set (younger presentations) contained presentations up to the age of approx. 30 years, whereas the second set (recent presentations) contained presentations starting from the age of approx. 40 years up to the age of approx. 60 years. There was a deliberate gap of ten years between the two sets to ensure the perceived facial difference was large enough.

Per set, images were mounted onto 24” displays that showed two images side by side. The distance to the center of the pictures was 15 cm. A chin rest was used to align the line of sight with the center of the display (with the distance from the monitor to the eyes being approx. 60 cm). For each model we generated displays of all possible combinations of images per model—not taking the lateralization effects of the displays into account—leading to 2 out of 8 combinations  =  28 displays for each model, so 4 [models] × 28 [displays per model]  =  112 combinatory displays for the entire set of single stimuli.

#### 4.1.3. Procedure

Participants were randomly assigned to one exposure condition of a model (e.g., Set 1 of model Female1956). Study 3 consisted of three phases across nine days (with a delay of 3 days between each phase) in a certain order: Phase 1: similarity rating  +  1 × deep evaluation; Phase 2: 3 × deep evaluation; Phase 3: 1 × deep evaluation  +  similarity rating (both sets)  +  prototypicality rating. For details, see Figure 13.

*Similarity rating*. In the first phase, similarly to Study 1, participants were asked to rate the *similarity* between both facial versions of one model on a 7-point Likert scale (ranging from 1  =  *very unsimilar* to 7  =  *very similar*). Each trial started with a fixation cross (presented for 500 ms) in the center of the screen, followed by a blank screen (presented for 100 ms), followed by a display of two facial depictions of one person which remained present until the participant made a response. This resulted in 2 out of 8 combinations  =  28 displays for each model. In the case of Phase 3, participants were presented with *all* presentations of both exposure conditions (younger presentations  +  recent presentations), resulting in 2 out of 16 [Set 1  +  Set 2] combinations  =  120 displays for each model (see Figure 13 for details). Participants were informed that the presented faces belong to the same person (“please note that all presented facial depictions belong to the very same person”).

*Deep evaluation*. In Phase 2, participants learned the faces with respect to their allocated exposure condition. Here we used the *Repeated Evaluation Technique* (RET) by Carbon and Leder (2005), which could be used for deep evaluation and familiarization of a learning set. This technique was further successfully used for e.g., priming, investigating mere exposure or adaptation studies in different scientific contexts (e.g., Carbon et al., 2008; Faerber et al., 2010; Muth & Carbon, 2013; Tinio & Leder, 2009). In this task, participants were repeatedly asked to rate stimuli across a large number of variables, thereby learning the presented stimuli. For the present study, we used the following evaluation variables, which mainly focus on facial features (using a 7-point Likert scale): *skin quality* (ranging from 1  =  *very unclear* to 7  =  *very clear*), *skin tone* (ranging from 1  =  *very dark* to 7  =  *very light*), *hair color* (ranging from 1  =  *very dark* to 7  =  *very light*), *hair length* (ranging from 1  =  *very short* to 7  =  *very long*), *eye distance* (ranging from 1  =  *very narrow* to 7  =  *very spaced apart*), *eye shape* (ranging from 1  =  *very narrow* to 7  =  *very round*) *eyebrow condition* (ranging from 1  =  *very thin* to 7  =  *very dense*), *facial salience* (by asking the participants “imagine that you see this person in group of other people. How salient would you rate the presented face?”; ranging from 1  =  *very unremarkable* to 7  =  *very remarkable*) and *sexual dimorphism* (ranging from 1  =  *very masculine* to 7  =  *very feminine*). The evaluation of the aforementioned variables was established blockwise in random order and was repeated three times but only at Phase 2, resulting in 8 [presentations] × 9 [variables]  =  72 trials for Phase 1 and Phase 3, but 8 [presentations] × 9 [variables] × 3 [repetitions]  =  216 trials for Phase 2. In total (across all phases), participants evaluated the presented faces over 72  +  216  =  288 trials.

*Prototypicality rating*. At the end of Phase 3, the prototypicality rating task was the same as in Study 2, except there were only 2 *episodic prototypes* (morphed exemplars of younger vs. recent depictions). As a result, we yielded 2 *episodic prototypes* (*younger prototype* and *recent prototype*)  +  1 *exhaustive prototype*  +  16 exemplars  =  19 facial exemplars per model set.

### 4.2. Results

Concerning the similarity ratings, statistical analyses were executed in the same style as in Study 1 with the exception that for external validation of the first step to automatically find the optimal number of clusters, we only invited five independent raters (4 female, *M*  =  22.3 years, *SD*  =  5.4) to find clusters on the basis of the plotted face-space (without information about the exposure date). All analyses focused on the difference between clusters of younger vs. recent exposure conditions *after* participants learned the respective faces. Accordingly, cluster analyses were applied to the data from Phase 3. In the following, all the results from the cluster analyses are presented per model. We further refrained from presenting detailed plots with respect to determining the best number of clusters. Instead, we will report the final results of these analyses.

#### 4.2.1. Model #1: Female, Born 1956 (Female1956)

**Figure 14.**
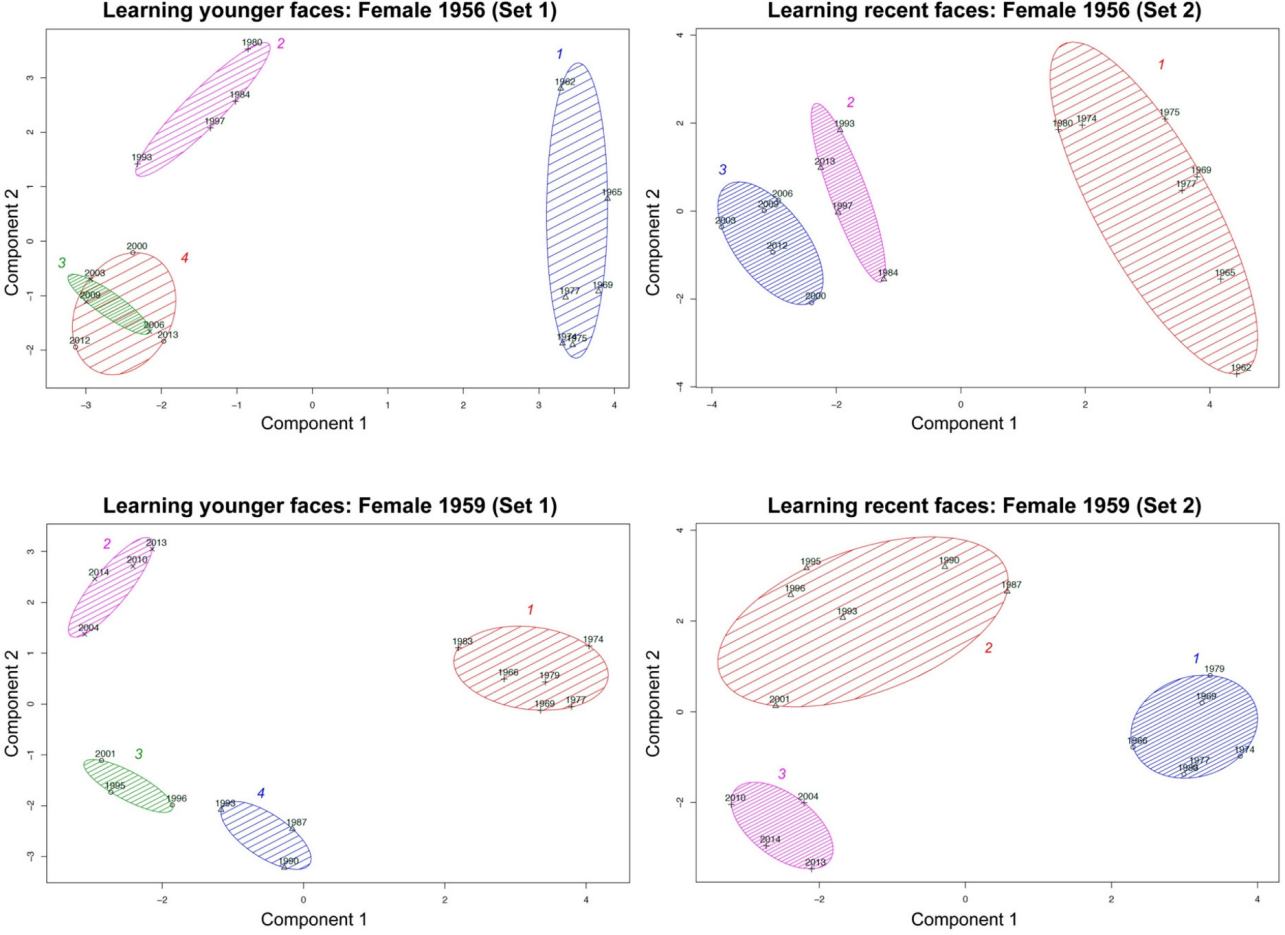
Upper row: Model #1: *Female1956*. Learning *younger* exemplars (Set 1) resulted in more and higher-graded clusters from recent decades, whereas learning *recent* exemplars (Set 2) resulted in three clusters with larger and lower graded recent clusters containing exemplars from different periods. Lower row: Model #2: *Female1959*: Results from cluster analyses for Model #2 were similar to Model #1. Learning *younger* exemplars led to more, and higher-graded clusters.

As shown in Figure 14 (upper row), analyses revealed clear sub-prototypical clusters in the outward facial appearance of the model, replicating the results of Study 1 that certain timespans reflect genuine *episodic prototypes*. Comparing the cluster solutions between the two exposure conditions (*younger presentations* vs. *recent presentations*), analyses revealed clear differences relating to the number of clusters as well as their configurations (e.g., cluster memberships). In the case of learning *younger presentations* (Set 1), exemplars of *recent* times were clustered in a higher resolution and constituted an additional cluster. In contrast, in the case of learning *recent presentations* (Set 2), exemplars of *recent* times were clustered in relatively large clusters followed by another in-homogeneous cluster containing exemplars from years in different decades (1984, 1993, 1997, and 2013)—see Figure 14. With regard to determining the optimal number of clusters, all criteria revealed the same results. Accordingly, the results of Model #1 provide the first hints that people who are (highly) familiar with *younger* exemplars and are then confronted with *recent* exemplars (only once) rapidly update their facial representation and build more highly graded sub-prototypes of *recent* times. They further perceive actually experienced (*younger*) facial exemplars as relatively unsimilar (suggested by the large gap between younger and recent exemplars).

#### 4.2.2. Model #2: Female, Born 1959 (Female1959)

As shown in Figure 14 (lower row), analyses revealed a similar pattern of clustering as for Model#1. Comparing the cluster solutions between the two exposure conditions (*younger presentations* vs. *recent presentations*), we replicated the differences relating to the number of clusters found for Model #1. Again, in the case of learning *younger presentations* (Set 1), exemplars of *recent* times were clustered in a higher resolution and constituted an additional cluster. In contrast to learning *recent presentations* (Set 2), exemplars of the last three decades were clustered in three very homogeneous clusters—see Figure 14. Here (Set 2), participants perceived these exemplars as a single relatively large cluster. With regard to determining the optimal number of clusters, all criteria revealed the same results for Set 1. In the case of Set 2, only the independent raters split the large cluster into two clusters.

#### 4.2.3. Model #3: Male, Born 1955 (Male1955)

**Figure 15.**
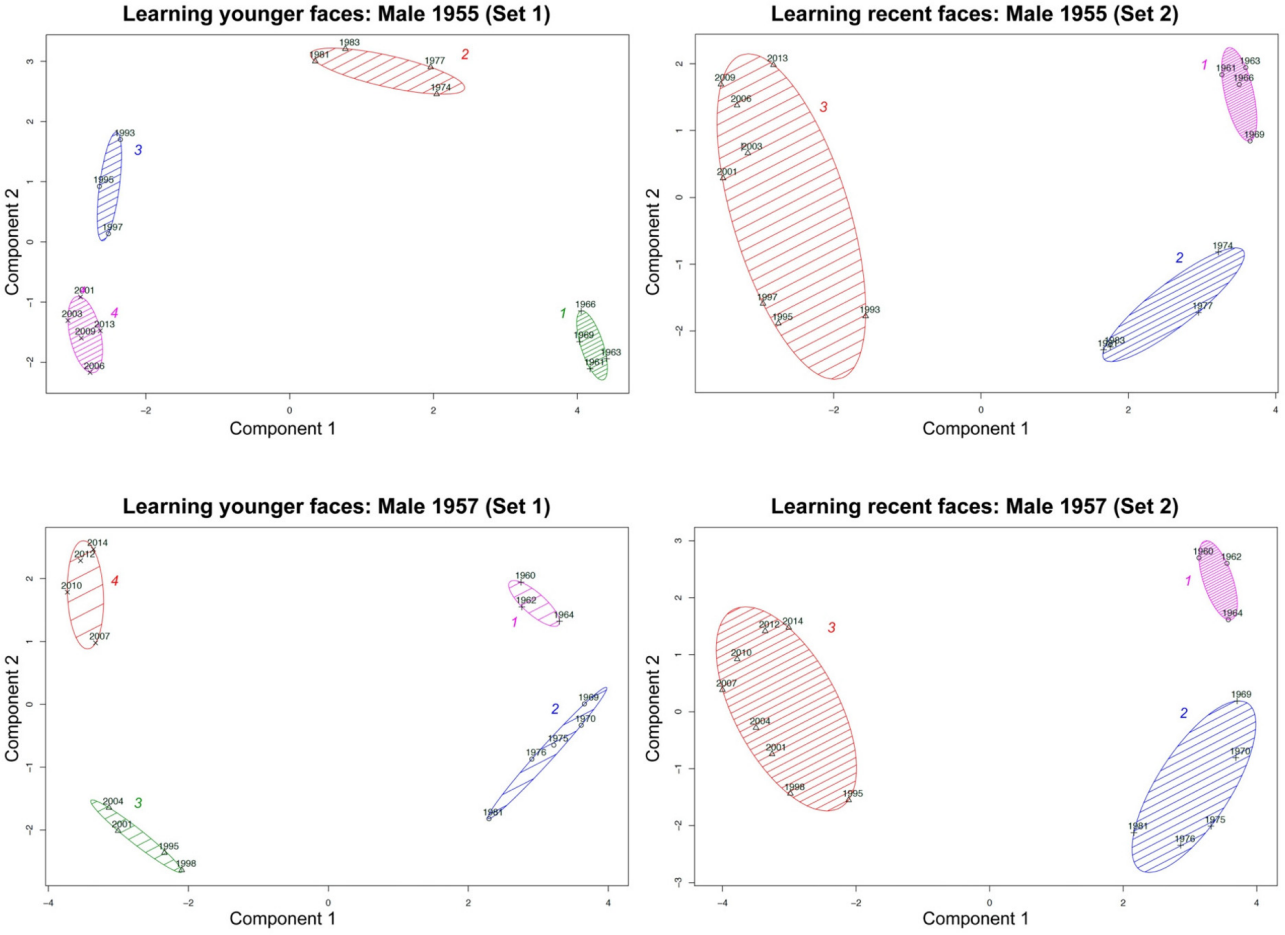
Upper row: Model #3: *Male1955*. Learning *younger* exemplars (Set 1) resulted in more and higher-graded clusters from recent decades, whereas learning *recent* exemplars (Set 2) resulted in three clusters with larger and lower graded recent clusters containing exemplars from different periods. Lower row: Model #4: *Male1957*: Results from cluster analyses for Model#2 were similar to Model#1. Learning *younger* exemplars led to more and higher graded clusters.

Results for Model #3 are shown in Figure 15 (upper row). Also, for the first male model, the results were similar to the female models. In the case of learning *younger presentations* (Set 1), exemplars of *recent* times were clustered in a higher resolution and constituted an additional cluster, whereas learning *recent presentations* (Set 2) led to one rather large cluster from the last three decades. Prima facie, a three-cluster solution for Set 2 may seem to be non-convincing. However, the gap statistic criterion, as well as the elbow criterion, suggested an appropriate number of three clusters. Only the human raters tended to choose a four-cluster solution (three out of five raters).

#### 4.2.4. Model #4: Male, Born 1957 (Male1957)

As shown in Figure 15 (lower row), there was again a clear tendency for finer-graded clusters of *recent* decades when participants learned *younger* faces (Set 1). When participants learned *younger* faces, there was a fourth additional cluster for depictions of recent episodes.

#### 4.2.5. Analyzing Perceived Prototypicality

**Figure 16.**
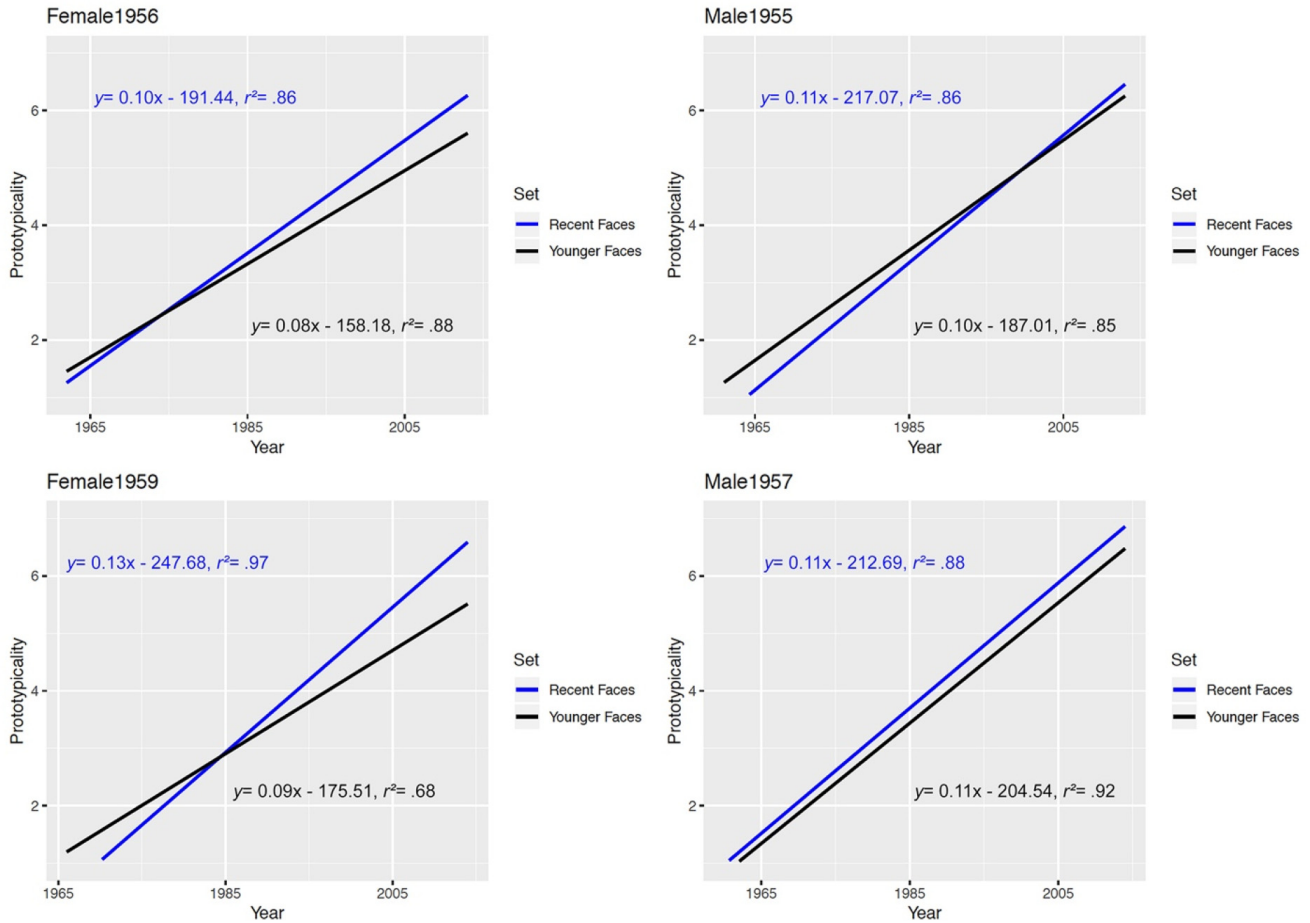
Linear regression analyses for the 4 [models] × 2 [exposure conditions/sets] conditions. Blue lines indicate regression lines for learned *recent* faces and black lines indicate the regression lines for learned *younger* faces. Participants of both exposure conditions perceived *recent* presentations as most prototypical, even when participants had only experienced *recent* presentations once-only (exposure condition *younger faces*).

We expected the genesis of facial prototypes to be based on adaptation mechanisms (e.g., Carbon and Ditye, 2011, 2012). Accordingly, exemplars that are experienced more frequently should be perceived as more prototypical. Similar to Study 2, we tested whether the *age* of the unique exemplars could predict the perceived prototypicality. Eight independent regression analyses (two per model: *younger* vs. *recent* faces) revealed that the predictor (independent variable) *age* satisfactorily predicted the perceived *prototypicality* (criterion)*;* see Figure 16 for details. This finding contrasts our hypothesis that there is adaptation towards the level of exposure (experiencing more *younger* vs. experiencing more *recent* faces). Furthermore, even if participants only learned *younger* faces, presentations from *recent* decades were perceived as most prototypical.

### 4.3. Discussion

We conducted Study 3 to investigate the process of facial learning concerning the individual level of experience. There are two main outcomes from this study. First, in line with the results of Study 1, we suggest that learned faces can be mentally organized in clearly genuine *temporal* clusters referring to certain EoL (what we call *episodic prototypes, EP*). In everyday life, learning faces is usually not a uniform process over time with equally distributed exposures to faces, as we might experience some more or less intense periods of familiarity with a person (and thus with her or his face)—we might even encounter full interruptions of interactions with persons (e.g., due to lacking personal contact to a person over the years), meaning we will encounter periods where no updated visual input related to the facial appearance of this person is available. In order to investigate such typical face-learning and memorization processes, we exposed participants to sets of faces relating to very different EoL (a youthful period vs. the most recent period of life). Interestingly, learning *younger* faces led to more finely graded *episodic prototypes* (in the sense that exemplars of an *episodic prototype* are perceived more similarly); in fact, it led to an additional *EP* from recent years. We have to cope with dramatic facial changes (e.g., due to the process of aging) and, based on deep elaboration of a young prototype, it seems that people then perceive more finely graded differences with more recent pictures. This specific result provides an important insight: The task in which a person is initially encountered with photos of a *younger* EoL simulates the learning of *how* a person's face ages across a certain lifespan. In fact, this requires the higher “resolution” of an *EP* in the sense that this process refers to a naturalistic prototype formation. In contrast, people who have learned more *recent* faces do not differentiate among them in detail but link them to one solid and long time-spanning cluster instead—it seems that they extract the essence of all these faces with all the useful variance provided by different depictions of a face (e.g., Burton et al., 2005; Burton et al., 2016; Ritchie and Burton, 2017; Young and Burton, 2017). Analyses revealed that the mere frequency of specific face presentations had no measurable impact on the perceived prototypicality. This is in contrast to the hypothesis that the process of prototype formation mainly relies on frequency-based adaptation (see e.g., Carbon and Ditye, 2011, 2012)—or on the general factor called “time” (Strobach & Carbon, 2013) or “adaptation duration” (Strobach et al., 2011), but is in accord with already existing data from other face learning paradigms (Schneider & Carbon, 2014). In situations where a face representation is deeply established by lifelong learning, e.g., in the typical case of relatives whom we have seen thousands of times in very different situations and under very different viewing conditions, we revealed a rapid update mechanism that is very effective in updating the mental representation instantly. This is quite surprising, as deeply established representations seem to need a kind of inertia of adaptation in order not to be overwritten too quickly. We have, however, documented such cases before, for instance in the case of a very well-established face, the portrait of Mona Lisa painted by Renaissance artist Leonardo da Vinci—this particular portrait is probably the best-known one in the world with thousands of expositions across life; nevertheless, a severe change to its facial configuration yields an instant change in the mental representation (Carbon & Leder, 2006). Such a rapid update mechanism was also documented for faces which were also mentally very well represented via a long learning history (Carbon et al., 2007)—an update of the prototype which lasted for a long period of time, e.g., one week (Carbon & Ditye, 2011), and persisted across different test settings (Carbon & Ditye, 2012). Actually, this potential risk of updating an established prototype too quickly via new visual inputs is offset by a potent mechanism to keep the mental representation up to date in order to be able to refer to the latest outward appearance of a known person. Such a mechanism is therefore particularly important for instances that refer to contemporary times but not to historical times where such a change does not occur.

## 5. Study 4

From the previous three studies, we provided evidence for the following three insights. First: We suggest *age* as a very potent factor for facial representations and prototypes. Even if we can mentally categorize faces along other variables such as facial expression, ethnicity, or gender (see e.g., Valentine, 1991; Valentine and Endo, 1992), regarding lifelong learning scenarios, *age* seems to have the strongest impact on the genesis of facial changes and so also on the genesis of facial representations and prototypes. Second: Recent presentations are perceived as being more prototypical, suggesting that the mental representation of a given face is adapted towards a more recent outward appearance of the respective identity. Third: We could also show that the frequency of exposure of a given face has *no* significant impact, contrasting evidence from classical frequency-based adaptation studies (see e.g., Carbon and Ditye, 2011, 2012). More in detail, *recent* presentations of a given face most likely correspond to one facial representation of a given identity (operationalized by perceived *prototypicality*). Accordingly, in contrast to simple unifying average-based prototypes models (see e.g., Burton et al., 2005; Jenkins and Burton, 2008; Valentine and Endo, 1992), we present the *Episodic Prototypes Model* (EPM) as a hybrid-model combining the strengths of average- *and* exemplar-based (e.g., Hintzman, 1984, 1986) approaches. More in detail, the *EPM* suggests that faces are represented in distinct temporal clusters, so-called *episodic prototypes* (*EPs*) reflecting the typical outward appearance of an episode of a human's life. Recent and more sophisticated approaches like the varying abstraction model (Vanpaemel & Storms, 2008) supports our idea of a partial abstraction rather than an *exhaustive* approach.

Finally, we claim that the *EPM* is more efficient and outperforms average-based approaches, which are commonly used in the research field of face perception. The previous three studies already provided first indications that we can reveal data pattern in a multitude of empirical paradigms which are compatible with Eps. Up to now, however, we lack rigorous experimental testing showing the recognition power of EPs. Accordingly, we conducted a fourth and final study to test this postulation by employing a face verification task utilizing reaction times (RTs) as a key measure.

### 5.1. Method

#### 5.1.1. Participants

Twenty-two persons participated in Study 4 (11 female; *M*  =  26.5 years, *SD*  =  7.1, range 20 to 51 years) on a voluntary basis. Most of the participants were undergraduate students of the University of Bamberg and gained course credit or twenty Euros to fulfill course requirements. All participants were naïve to the aim of the study and were not familiar with the presented faces. They were all assessed as being normal in terms of visual acuity tested via a standard Snellen Eye chart test. Color-vision for all participants was ensured by testing them via a self-made short version of the Ishihara color test. All participants gave written consent to participate in the study. All procedures and treatments of participants were in accordance with the Declaration of Helsinki. The study was in full accordance with the ethical guidelines of the University of Bamberg and was approved by the University Ethics Committee on 18 August 2017.

#### 5.1.2. Materials

We used the same material as in Study 1 but with the major difference that we pre-processed all images by reducing color saturation (chroma), adding a sepia picture plane (30% opacity) and slight blur in case of higher focus depth at the same level *plus* adding slight scratches using Adobe Photoshop CC 2021. Some older depictions, especially from the 1970s and 1980s were excluded from this procedure since this procedure would has added to much distortion. The main idea behind this approach was to make the material visually more homogenous and *reduce* available visual information such as the picture quality what can be used to make inferences about the actual age of the picture. Please note that we did not aimed to eliminate any and all potential sources of biases such as passing fad or hairstyle what also might offer information to make inferences about the actual age of the depicted individual. However, most of the participants from the first three studies stated that the perceived age of the depicted individual had the highest impact on their decisions.

**Figure 17.**
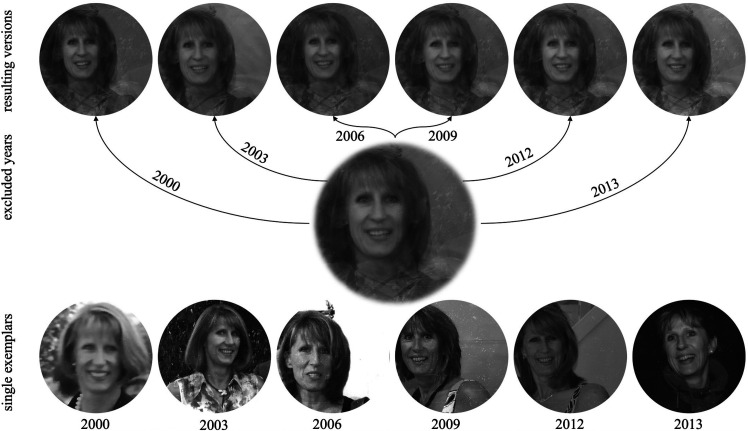
Demonstration of the exclusion procedure regarding *Female1959*. Bottom line: single exemplars of the 2000s’ *EP* (in the middle). We stepwise excluded every single exemplar what resulted in six versions (upper line). For example, the first resulting version consisted of the exemplars from the years 2003, 2006, 2009, 2012, and 2013 (2000 was excluded).

Subsequently, we created morphs (*EPs*) for each decade of the model's life (1960s, 1970s, 1980s, 1990s, and 2000s) using the photo-morphing software Abrosoft^©^ FantaMorph V5 (5.4.6) and the same morphing procedure as for Study 2. For each *EP*, we excluded the target exemplar from the total number of exemplars (‘drawn without placing back’/’choosing without replacement’). For example, in case of model *Female1959*, the set for the 2000s *EP* was based on five exemplars from the years 2000, 2003, 2006, 2009, 2012, and 2013, so for a 2013 target we excluded that 2013 exemplar from the respective *EP*. This resulted in six versions of this *EP* so that each version consisted only of five single exemplars (see Figure 17 for an example). Please note that not every *EP* consisted of the same number of single exemplars. On average, each *EP* consisted of 3.5 single exemplars. However, the *EP* of the 2000s consisted of 6 single exemplars on average. We applied the same procedure to all *exhaustive prototypes* (comprising all 20 single exemplars per model). For example, in the case of the *exhaustive prototype* of model *Female1956* (consisting of all twenty exemplars), we created twenty versions of this *exhaustive prototype* where for each version one single exemplar was excluded. The main idea behind this approach was to avoid using the same stimulus material (with no sufficient variance) but still having enough trials for valid reaction times measurements. Consequently, we yielded 70 versions of *episodic prototypes* (1960s, 1970s, 1980s, 1990s, and 2000s *episodic prototypes*)  +  80 versions of *exhaustive prototypes*  =  150 presentations in total.

#### 5.1.3. Procedure

Study 4 was implemented in two steps. In a first step, participants were asked to learn the faces applying the same deep evaluation technique as used in Study 3 with two important differences: First, we also provided a fictional name for each model (*Female1956* was named *Elke*, *Female1959* was named *Gabi*, *Male1955* was named *Theo*, and *Male1959* was named *Luka*) above each presented face (e.g., “*This is Elke…*”). Second, we used the same nine evaluation variables as in Study 3 (*skin quality*, *skin tone*, *hair color, hair length, eye distance*, *eye shape, eyebrow condition*, *facial salience*, and *sexual dimorphism*) but added *chin form* and *head form* (both ranging from 1  =  *very narrow* to 7  =  *very round*) to also consider rather external face features—this should ensure elaboration of the faces to a maximum of visual properties. The evaluation of the aforementioned variables was established in random order resulting in 4 × [models] × 20 [presentations] × 11 [variables]  =  880 trials regarding the learning procedure.

The second step of Study 4 was a face verification task. All 150 versions of *episodic prototypes*  +  *exhaustive prototypes* were presented in the middle of a 24” display in random order. Participants were asked to decide whether there was a match between name and the presented face or not as fast as possible by providing them with the question, e.g., “*Is this Elke?*” right above each face. Combinations between faces and names were counterbalanced within the model's sex. We recorded *reaction times* (RT) and the quality of the response (correct vs. incorrect). Each trial started with a fixation cross (200 ms)  +  blank screen (200 ms) followed by the target until a response was made by the participant on the keyboard. With respect to the learning procedure, participants could take a short break after every 88 trials. The whole procedure of Study 4 lasted approx. 75 min.

### 5.2. Results

#### 5.2.1 Data Analysis Strategy

The data was processed using R 4.0.4 (R Core Team, 2013). In addition to the lme4 package (Bates et al., 2015) to perform linear mixed-effects analyses, R packages lmerTest (Kuznetsova et al., 2017) and ggplot2 (Wickham, 2016) were mainly used during the analysis of the data.

#### 5.2.2. Data Analysis

The main hypothesis which we tested was that episodic prototypes are the more adequate format to represent facial prototypes than exhaustive prototypes comprising all singular facial exemplars of a person. We operationalized this by a more efficient usage of episodic prototype indicated by faster reaction times (RTs) in a face verification task. Before we conducted analyses on the RTs, we tested for differences between the exhaustive and the EP models regarding correctness data to be sure that RTs are not biased by a speed-accuracy tradeoff. This was done, like all following statistical analyses, by means of utilizing Linear Mixed Models (LMM). We always followed a subsequent testing strategy with increasing complexities of the employed models. Therefore, we first defined a null model (Model #0) with factors involved for which we had no specific hypothesis in mind: Model #0 used no fixed factors, but only two random factors: *CaseID* (the participant ID) and *Model* (depicted person: the four depicted persons, called “models”). For the respective subsequent model, we added only one factor at a time to be able to via likelihood ratio tests which model was the most adequate model concerning the degree of fitting while being still parsimonious. Each model's residuals were visually inspected to exclude models deviating from homoscedasticity or normality.

**Figure 18.**
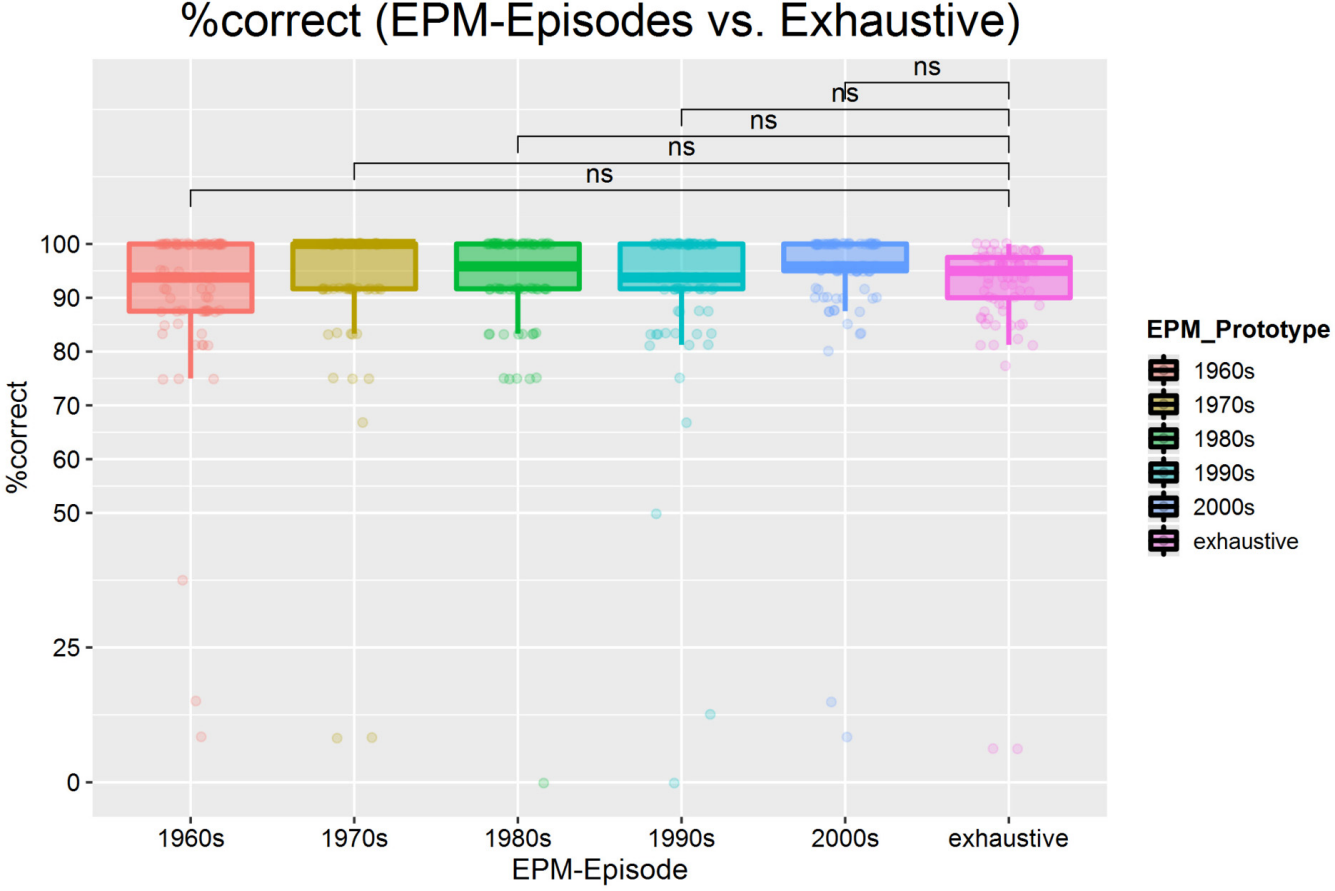
Accuracy values (in percent) for the different Episodic Prototype Model (EPM) Episodes and the Exhaustive Prototype. Boxplots along with individual data points show the distribution of matching accuracy. No significant differences between EPM Prototypes and the Exhaustive Prototype were found on a level of *p* < .05.

To test the correctness data (*M* = 92.1%, see Figure 18 for details) for differences between the general conditions of verifying a person on the basis of an exhaustive vs. episodic prototype, we tested the Null Model for correctness against Model #1 for which we added the general prototype model as fixed factor. The likelihood ratio test between Model#1 and the Null Modell yielded no significant result (see Table 2) indicating no further predictive power of adding the general prototype model, so a speed-accuracy effect could not be revealed.

**Table 2.** Comparison of models for the dependent variables correctness and reaction times (RT) of correct responses. *N_par_*  =  number of model's parameters, *AIC*  =  Akaike information criterion, an estimator of prediction error, −2LL  =  likelihood ratio, *df*  =  degrees of freedom and *p*  *=*  *p*-value of the regarding χ^2^-test (comparing the present model with the preceding one, e.g., the columns for Model #1 indicate the comparison between Model #1 and Model #0).

Models for *CORRECTNESS*	*N_par_*	*AIC*	−2LL	*df*	χ^2^	*p*
**#0: 1 + (1 ∣ MODEL) + (1 ∣ CASEID)**	4	122,717	−61,355			
**#1: 1 + PROTOTYPE + (1 ∣ MODEL) + (1 ∣ CASEID)**	5	122,717	−61,355	1	2.5	.1161
Models for *RT* *(CORRECT)*	***N_par_***	***AIC***	**−2LL**	***df***	**χ^2^**	***p***
**#0: 1 + (1 ∣ MODEL) + (1 ∣ CASEID)**	4	158,783	−79,387			
**#1: 1 + PROTOTYPE + (1 ∣ MODEL) + (1 ∣ CASEID)**	5	157,055	−78,522	1	1,730.2	<.0001
**#2: 1 + EPM_PROTOTYPE + (1 ∣ MODEL) + (1 ∣ CASEID)**	9	156,831	−78,456	4	132.0	<.0001

Based on this, we further tested for effects of the specific prototype used on the efficiency of the face verification process on the basis of reaction time data for correct responses. We first excluded RT outliers on the following criterion: RTs (of correct responses) which were faster than 200 ms and above 2.5 *SD*s of the individual mean of RTs were interpreted as RT outliers. This very conservative criterion resulted in a data loss of 1.1%.

Table 2 shows the subsequent testing of models (Model #1 again used the general prototype model as fixed factor, Model #2 used the more fine-graded specific episodic prototypes compared with the exhaustive prototype) resulting in identifying Model #2 as the most adequate model to describe the data, explaining 47.9% of the variance of the data—details on the further outcome of this model can be retrieved from Table 3.

**Table 3.** Linear mixed model #2 identified as most adequate to describe the reaction time (RT) data pattern by subsequent testing of model #1 against model #0 and then model #2 against model #1 via likelihood ratio tests. Bold numbers show significant results.

RT (correct)			
	Model2		
*Predictors*	*Estimates*	*p*	*df*
(Intercept)	790.80 ***	<0.001	12,006.00
exhaustive	*Reference*		
1960s	−96.88 ***	<0.001	12,006.00
1970s	−113.57 ***	<0.001	12,006.00
1980s	−117.42 ***	<0.001	12,006.00
1990s	−139.23 ***	<0.001	12,006.00
2000s	−161.68 ***	<0.001	12,006.00
*ICC*	0.43		
*N*_Model_	4		
*N*_SNr_	22		
Observations	12,015		
Marginal *R*^2^ / Conditional *R*^2^	0.088 / 0.479		
*AIC*	156,930.712		
log-Likelihood	−78,456.356		
*** p *<* *0.001*			

**Figure 19.**
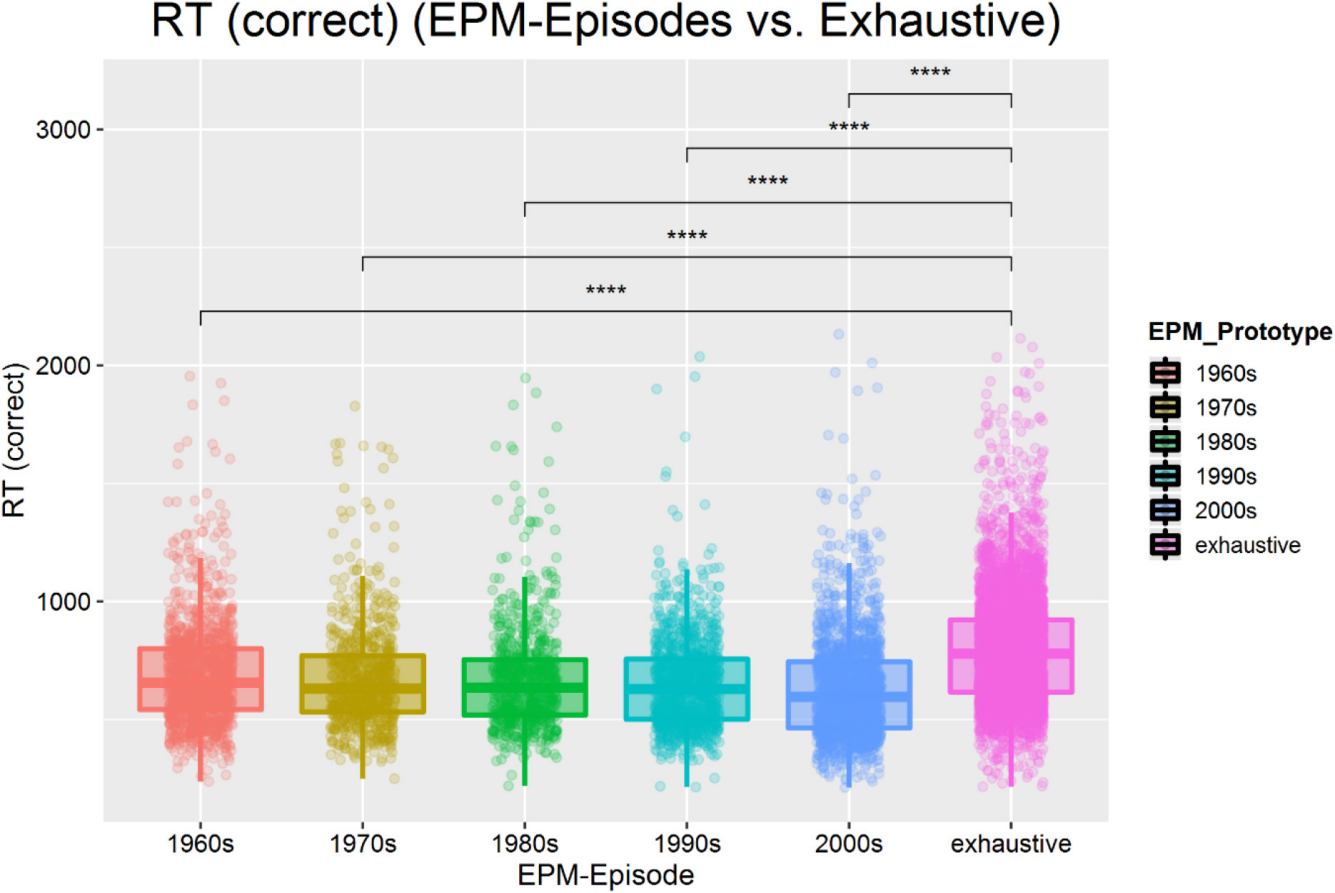
Reaction times (RT) for the different Episodic Prototype Model (EPM) Episodes and the Exhaustive Prototype. Boxplots along with individual data points show the distribution of RTs. Statistical tests are based on the LMM approach shown in Table 3. All differences between EPM Prototypes and the Exhaustive Prototype were found significant on a level of *p* < .0001.

As can be seen in Figure 19 (and which can be statistically retrieved from respective Table 3 about the LMM testing), all episodic prototypes were faster processed than the exhaustive prototype.

### 5.3. Discussion

Evidence from the first three studies demonstrates the strengths of the *EPM* in contrast to standard exhaustive prototype models. Study 4 was conducted to investigate the postulated efficiency and advantages of this approach. We could show that *all* episodic prototypes were processed significantly faster than a given exhaustive prototype. This effect was remarkably large and yield an average benefit of reaction times in the range of 96 to 161 ms (see Table 3). Importantly, this effect was also very robust, as shown by similar effects across the participants. As can be retrieved from Figure 19, not only the face verification was slowed down when exhaustive prototypes were used instead of episodic prototypes, shown by significant effects of the exhaustive prototype against any of the episodic prototypes, but there was also a further, seemingly hierarchical, effect of episodic prototypes: The more recent an episodic prototype was, or put it differently: the closer an episodic prototype was to the latest instances of an outward facial appearance, the faster a verification task could be executed. This indicates that not only the mental representation of faces in terms of episodic prototypes is beneficial, but that learning many versions of faces coming with an aging component seemingly leads to a chronologically ordered relevance of episodic prototypes. And as long as we rely on evolutionary-shaped cognitive mechanisms which evolved over a very long time of phylogenesis where clear-cut references to bygone times were not available, the latest representatives, compiled together to the most recent episode of life, is the most important mental representation as it is most indicative for successful face verification of versions similar to the current outward appearance. Such a noticeable side finding that was not in the focus of our present work might be most interesting for consequently developing a representational memory model based on facial episodes further.

## 6. General Discussion

The main goal of the present study was to investigate the nature of facial representations and the genesis of facial prototypes with a focus on temporal dynamics in facial development. In contrast to classical *average models* (e.g., Burton et al., 2005; Franks and Bransford, 1971; Gao and Wilson, 2014) of mental representations, which postulate that prototypes are *abstractions* of a certain concept as a result of *averaging characteristic features,* we postulate the *Episodic Prototypes Model,* which seems to be more economical while preserving the more typical characteristics of a face.

In accordance with well-established *exemplar-based models* (e.g., the *MINERVA-2* model by Hintzman, 1984, 1986), we could demonstrate that the assumption of a particular *exhaustive prototype—*which should correspond to the facial representation of a particular person (see, e.g., Benson and Perrett, 1993; Busey, 1998; Reinitz et al., 1992; Webster et al., 2004; Webster and MacLeod, 2011) for a long-term life phase or even the whole lifetime—is sub-optimal. In fact, an *exhaustive prototype* cannot represent the typical characteristics of a face. Recent studies have investigated the process of how a face becomes familiar and have revealed that learning a new face involves an abstraction of the *variability* of different images belonging to the very same person's face (see e.g., Burton et al., 2016; Kramer et al., 2017; Matthews et al., 2018; Menon et al., 2015; Murphy et al., 2015; Ritchie et al., 2018; Ritchie & Burton, 2017; White et al., 2014). However, the process of learning a face could not be sufficiently described by such temporal-invariant models. Although these recent approaches are more sophisticated than first-generation prototype models from the 1990s (e.g., Valentine, 1991; Valentine and Bruce, 1986; Valentine and Endo, 1992), they do not provide any decisive information about how faces are mentally represented (e.g., average-based vs. exemplar-based) or whether *temporal* aspects have a significant impact on the genesis and representation of facial prototypes. The findings of the present study highlight a very critical problem in this respect. Although we have gained a varied body of information on *how* faces are recognized (e.g., Akselrod-Ballin & Ullman, 2008; Bindemann et al., 2010; Bruce & Young, 1986; Burton et al., 2005; Carbon, 2008; Wirth & Carbon, 2017), face research still lacks knowledge and theories on *which* variables (e.g., *temporal*) are essential for generating facial prototypes and representations. Most research still does not deal with the variability of outward facial appearances. Furthermore, the specific process of learning is hardly a topic for research. Real-life experiences and learning in real-life conditions have been covered even less by recent approaches. All these methods yield relatively simple models of facial representations, which typically refer to *average* or *exhaustive* prototypes. An *averaged representation* produces and *average “*echo” (in the sense of Hintzman's 1984, *MINERVA-2 Model*)—see Figure 12—and thus provides a very economical solution to a simple representational basis which certainly outperforms mere single exemplar-based approaches (see e.g., Busey, 1998; Valentine and Bruce, 1986; Valentine and Endo, 1992). Furthermore, facial representations could be described as abstractions of *all* the characteristic attributes of respective faces (e.g., Franks and Bransford, 1971). It is not surprising that *averaged* representations of a person's face are more reliably and quickly recognized than single exemplars, which are used in passports (e.g., Benson and Perrett, 1993; Burton et al., 2005; Busey, 1998; Gao and Wilson, 2014; Jenkins and Burton, 2008; Reinitz et al., 1992; Webster et al., 2004; Webster and MacLeod, 2011). If the timespan to be integrated is too long, however, we face a striking loss of variance for such singular prototypes.

In the present study, we demonstrated that this inherent problem could be avoided by assuming a model that is much closer to the typical learning histories of faces. The *Episodic Prototype Model (EPM)* explicitly deals with the typically increasing variability of facial representations over time (Figure 12) by employing several prototypes of a given face that represent different EoL. Such prototypes were indeed revealed by all three studies. Furthermore, we showed that the facial representation of a respective person is biased toward recent experiences when participants experienced a larger number of younger presentations. At first glance, this result seems to be in contrast to the *attribute-frequency model* (see e.g., Neumann, 1974, 1977; Solso and McCarthy, 1981b), which postulates a prototype as a pattern that incorporates the most frequently experienced feature within each attribute. Our results showed that this model remains valid for specific and limited timespans in extreme cases, such as are simulated in Study 3, we suggest that it is more economical for the cognitive system to perform an almost instant update of the facial representation what is plausible as we have to cope rapidly with dramatic facial changes after a long period of time. This postulation is supported by recent research, which revealed that the level of experience (frequency of exposure) has *no* impact on the perceived prototypicality (Schneider & Carbon, 2014). In contrast, it could be shown that *familiarity* is a more important predictor for prototypicality. Accordingly, we suggest that we perceive *recent* presentations as more *familiar*, as we experience the respective person in the *present* (and not in the past). Finally, we tested in Study 4 our major claims of the *EPM* by using a well-established approach in face research (see e.g., Bruce & Young, 1986; Burton et al., 2005; Thorpe, Fize, & Marlot, 1996). We used a face verification task and revealed that *episodic prototypes* are significantly faster processed than exhaustive prototypes supporting the idea that the *EPM* is more efficient and outperforms average-based approaches, which are commonly suggested to be good candidates for robust face representations (see e.g., Burton et al., 2005).

We would also like to make clear some limitations of our present study. First, the explicit usage of authentic and highly idiosyncratic stimulus material made it rather hard to access professional and standardized photo material. However, we also want to stress that recent research (Schneider & Carbon, 2017a) indeed used highly professional and standardized stimulus material and still found similar *temporal* cluster configurations, being in accordance with the *EPM* described here. Thus, we expect that the stimulus material in the present study was of sufficient quality and reflected the typical material we assess in everyday life. Second, with respect to the so-called *representation enhancement hypothesis,* there is some evidence that faces which were learned in motion can be recognized more accurately than learned from static views (see e.g., Lander et al., 1999; Lander & Bruce, 2000, 2003; Lander & Chuang, 2005; O'Toole & Roark, 2011). Accordingly, one could suggest that facial representations are stored in motion. For Study 2, a large number of the participants (who were relatives of the respective model) stated that they found the task very difficult since they typically encountered the persons in real-life events in a dynamic and interactive way. This fact could be crucial for further research into facial prototypes and representations. As mentioned in the introduction, there is only sparse knowledge on how the mental representation of a face is built up and how exactly faces are further represented. This process, again, could be mainly influenced by temporal as well as contextual variables (e.g., there is an “*always-smiling”* relative whose prototype has a variety of smiling and moving facial expressions). This leads to another important question that was considered before: how do multimodal information and social interactions play a role in prototype formation?

With respect to the multidimensional face-space, past research has always been content to extend this face-space with an additional and abstracted *temporal* dimension in order to describe temporal aspects. The utility of such explicit and rather abstracted dimensions seems quite limited, as it does not reflect the individually based face-space with respect to the aforementioned idiosyncratic and interactive temporal facial development. However, one important question remains: is age only one out of several variables that affects the genesis of facial prototypes and presentations, or does it point to the core of the whole generation principle of new episodic prototypes? Certainly, we would not disagree with the idea that prototype formation may rely on aspects other than aging (e.g., changing facial expression, fashion style—principally hairstyle—or most of all skin tone due to a severe health problem) see e.g., Abudarham et al. (2019) and Gotlieb et al. (2020). However, in accordance with recent research (e.g., Mileva et al., 2020), we propose that time is presumably the most important variable since almost all facial changes across the whole lifetime are age-related or age-induced.

We hope that our *Episodic Prototypes Model* (EPM), together with the accompanying empirical studies, contributes to the understanding of how facial representations are generated and retrieved. The postulated EPM aims to further specify the nature of so-called face-spaces by propagating an episodic view on facial representations. This might help to better understand why we are on the one hand often so brilliant at recognizing familiar persons based on just one single image that fits with experienced episodes of such a person, but why on the other hand we are often poor at identifying the very same person from lots of images which stem from other, less or not-experienced episodes. Further research should investigate potentially interesting additions to the here developed EPM such as individually weighted prototypes or specifically defined facial information, which lead to more effective changes in prototypes. We would be very happy to see our preliminary approach inspiring more work on this important field of face research.

## References

[bibr1-20416695211054105] Abudarham N. Shkiller L. Yovel G. (2019). Critical features for face recognition. Cognition, 182, 73–83. 10.1016/j.cognition.2018.09.002 30218914

[bibr2-20416695211054105] Akselrod-Ballin A. Ullman S. (2008). Distinctive and compact features. Image and Vision Computing, 26(9), 1269–1276. 10.1016/j.imavis.2008.03.005

[bibr3-20416695211054105] Azizi E. Lusky A. Kushelevsky A. P. Schewachmillet M. (1988). Skin type, hair color, and freckles are predictors of decreased minimal erythema ultraviolet-radiation dose. Journal of the American Academy of Dermatology, 19(1), 32–38. 10.1016/S0190-9622(88)70148-6 3403743

[bibr4-20416695211054105] Basri R. (1996). Recognition by prototypes. International Journal of Computer Vision, 19(2), 147–167. 10.1007/BF00055802

[bibr5-20416695211054105] Bates D. Mächler M. Bolker B. Walker S. (2015). Fitting Linear Mixed-Effects Models Using lme4. Journal of Statistical Software, 67(1), 1–48. 10.18637/jss.v067.i01

[bibr6-20416695211054105] Benson P. J. Perrett D. I. (1993). Extracting prototypical facial images from exemplars. Perception, 22(3), 257–262. 10.1068/p220257 8316513

[bibr7-20416695211054105] Bindemann M. Avetisyan M. Blackwell K. A. (2010). Finding needles in haystacks: Identity mismatch frequency and facial identity verification. Journal of Experimental Psychology: Applied, 16(4), 378–386. 10.1037/a0021893 21198254

[bibr8-20416695211054105] Brooks L. R. (1978). Nonanalytic concept formation and memory for instances. In Rosch E. Lloyd B. (Eds.), Cognition and categorization (pp. 169–211). Elbaum.

[bibr9-20416695211054105] Bruce V. Young A. (1986). Understanding face recognition. British Journal of Psychology, 77, 305–327. 10.1111/j.2044-8295.1986.tb02199.x 3756376

[bibr10-20416695211054105] Bruck M. Cavanagh P. Ceci S. J. (1991). Fortysomething - recognizing faces at ones 25th reunion. Memory & Cognition, 19(3), 221–228. https://doi.org/0.3758/Bf03211146 1861608 10.3758/bf03211146

[bibr11-20416695211054105] Burt D. M. Perrett D. I. (1997). Perceptual asymmetries in judgements of facial attractiveness, age, gender, speech and expression. Neuropsychologia, 35(5), 685–693. 10.1016/S0028-3932(96)00111-X 9153031

[bibr12-20416695211054105] Burton A. M. Jenkins R. Hancock P. J. B. White D. (2005). Robust representations for face recognition: The power of averages. Cognitive Psychology, 51(3), 256–284. 10.1016/j.cogpsych.2005.06.003 16198327

[bibr13-20416695211054105] Burton A. M. Kramer R. S. S. Ritchie K. L. Jenkins R. (2016). Identity from variation: Representations of faces derived from multiple instances. Cognitive Science, 40(1), 202–223. 10.1111/cogs.12231 25824013

[bibr14-20416695211054105] Busey T. A. (1998). Physical and psychological representations of faces: Evidence from morphing. Psychological Science, 9(6), 476–483. 10.1111/1467-9280.00088

[bibr15-20416695211054105] Carbon C. C. (2008). Famous faces as icons: The illusion of being an expert in the recognition of famous faces. Perception, 37(5), 801–806. 10.1068/p5789 18605151

[bibr16-20416695211054105] Carbon C. C. (2009). What “exactly” is a prototype? Not sure, but average objects are not necessarily good candidates for. Journal of Vision, 9(8), 512–512. 10.1167/9.8.512

[bibr17-20416695211054105] Carbon C. C. (2011). The first 100 milliseconds of a face: On the microgenesis of early face processing. Perceptual and Motor Skills, 113(3), 859–874. 10.2466/07.17.22.Pms.113.6.859-874 22403930

[bibr18-20416695211054105] Carbon C. C. Ditye T. (2011). Sustained effects of adaptation on the perception of familiar faces. Journal of Experimental Psychology: Human Perception and Performance, 37(3), 615–625. 10.1037/a0019949 20731521

[bibr19-20416695211054105] Carbon C. C. Ditye T. (2012). Face adaptation effects show strong and long-lasting transfer from lab to more ecological contexts. Frontiers in Psychology, 3(3), 1–6. 10.3389/fpsyg.2012.00003 22291676 PMC3264890

[bibr20-20416695211054105] Carbon C. C. Leder H. (2005). The repeated evaluation technique (RET). A method to capture dynamic effects of innovativeness and attractiveness. Applied Cognitive Psychology, 19(5), 587–601. 10.1002/acp.1098

[bibr21-20416695211054105] Carbon C. C. Leder H. (2006). The Mona lisa effect: Is ‘our’ lisa fame or fake? Perception, 35(3), 411–414. 10.1068/p5452 16619955

[bibr22-20416695211054105] Carbon C. C. Michael L. Leder H. (2008). Design evaluation by combination of repeated evaluation technique and measurement of electrodermal activity. Research in Engineering Design, 19(2-3), 143–149. 10.1007/s00163-008-0045-2

[bibr23-20416695211054105] Carbon C. C. Strobach T. Langton S. R. H. Harsanyi G. Leder H. Kovacs G. (2007). Adaptation effects of highly familiar faces: Immediate and long lasting. Memory & Cognition, 35(8), 1966–1976. 10.3758/Bf03192929 18265612

[bibr24-20416695211054105] Charrad M. Ghazzali N. Boiteau V. Niknafs A. (2014). Nbclust: An R package for determining the relevant number of clusters in a data set. Journal of Statistical Software, 61(6), 1–36. 10.18637/jss.v061.i06

[bibr25-20416695211054105] Coetzee V. Chen J. Y. Perrett D. I. Stephen I. D. (2010). Deciphering faces: Quantifiable visual cues to weight. Perception, 39(1), 51–61. 10.1068/p6560 20301846

[bibr26-20416695211054105] Coetzee V. Perrett D. I. Stephen I. D. (2009). Facial adiposity: A cue to health? Perception, 38(11), 1700–1711. 10.1068/p6423 20120267

[bibr27-20416695211054105] Davies G. M. Ellis H. D. Shepherd J. W. (1981). Perceiving and remembering faces. Academic Press.

[bibr28-20416695211054105] Daw N. Courville A. (2008). The pigeon as particle filter. In Platt J. C. Koller D. Singer Y. S R. (Eds.), Advances in neural information processing systems 20 (pp. 369–379). MIT Press.

[bibr29-20416695211054105] Faerber S. J. Kaufmann J. M. Leder H. Martin E. M. Schweinberger S. R. (2016). The role of familiarity for representations in norm-based face space. Plos One, 11(5). 10.1371/journal.pone.0155380 PMC4864226 27168323

[bibr30-20416695211054105] Faerber S. J. Leder H. Gerger G. Carbon C. C. (2010). Priming semantic concepts affects the dynamics of aesthetic appreciation. Acta Psychologica, 135(2), 191–200. 10.1016/j.actpsy.2010.06.006 20615491

[bibr31-20416695211054105] Franks J. J. Bransford J. D. (1971). Abstraction of visual patterns. Journal of Experimental Psychology, 90(1), 65–74. 10.1037/h0031349 5096129

[bibr32-20416695211054105] Gao X. Q. Wilson H. R. (2014). Implicit learning of geometric eigenfaces. Vision Research, 99(0), 12–18. 10.1016/j.visres.2013.07.015 23911769

[bibr33-20416695211054105] Gershman S. J. Monfils M. H. Norman K. A. Niv Y. (2017). The computational nature of memory modification. Elife, 6, 10.7554/eLife.23763 PMC5391211 28294944

[bibr34-20416695211054105] Gershman S. J. Radulescu A. Norman K. A. Niv Y. (2014). Statistical computations underlying the dynamics of memory updating. Plos Computational Biology, 10(11). 10.1371/journal.pcbi.1003939 PMC4222636 25375816

[bibr35-20416695211054105] Girden E. R. (1992). ANOVA: Repeated measures. Sage Publications.

[bibr36-20416695211054105] Gotlieb M. Abudarham N. Shir Y. Yovel G. (2020). *Conceptual rather than perceptual similarity enables generalization across perceptually different appearances of familiarized faces*. https://doi.org/10.31234/osf.io/p6d5j.

[bibr37-20416695211054105] Hancock P. J. B. Bruce V. Burton A. M. (2000). Recognition of unfamiliar faces. Trends in Cognitive Sciences, 4(9), 330–337. 10.1016/S1364-6613(00)01519-9 10962614

[bibr38-20416695211054105] Hintzman D. L. (1984). Minerva-2: A simulation-model of human-memory. Behavior Research Methods Instruments & Computers, 16(2), 96–101. 10.3758/BF03202365

[bibr39-20416695211054105] Hintzman D. L. (1986). Schema abstraction in a multiple-trace memory model. Psychological Review, 93(4), 411–428. 10.1037//0033-295x.93.4.411

[bibr40-20416695211054105] Huynh H. Feldt L. S. (1976). Estimation of the Box correction for degrees of freedom from sample data in randomized block and split-plot designs. Journal of Educational and Behavioral Statistics, 1(1), 69–82. 10.3102/10769986001001069

[bibr41-20416695211054105] Jenkins R. Burton A. M. (2008). 100% Accuracy in automatic face recognition. Science (new York, N Y ), 319(5862), 435–435. 10.1126/science.1149656 18218889

[bibr42-20416695211054105] Jenkins R. White D. Van Montfort X. Burton A. M. (2011). Variability in photos of the same face. Cognition, 121(3), 313–323. 10.1016/j.cognition.2011.08.001 21890124

[bibr43-20416695211054105] Johnston R. A. Kanazawa M. Kato T. Oda M. (1997a). Exploring the structure of multidimensional face-space: The effects of age and gender. Visual Cognition, 4(1), 39–57. 10.1080/713756750

[bibr44-20416695211054105] Johnston R. A. Milne A. B. Williams C. Hosie J. (1997b). Do distinctive faces come from outer space? An investigation of the status of a multidimensional face-space. Visual Cognition, 4(1), 59–67. 10.1080/713756748

[bibr45-20416695211054105] Kramer R. S. S. Manesi Z. Towler A. Reynolds M. G. Burton A. M. (2017). Familiarity and within-person facial variability: The importance of the internal and external features. Perception, 47(1), 3–15. 10.1177/0301006617725242 28803526

[bibr46-20416695211054105] Kuznetsova A. Brockhoff P. B. Christensen R. H. B. (2017). lmerTest Package: Tests in Linear Mixed Effects Models. Journal of Statistical Software, 82(13), 1–26. 10.18637/jss.v082.i13

[bibr47-20416695211054105] Lander K. Bruce V. (2000). Recognizing famous faces: Exploring the benefits of facial motion. Ecological Psychology, 12(4), 259–272. 10.1207/S15326969eco1204_01

[bibr48-20416695211054105] Lander K. Bruce V. (2003). The role of motion in learning new faces. Visual Cognition, 10(8), 897–912. 10.1080/13506280344000149

[bibr49-20416695211054105] Lander K. Christie F. Bruce V. (1999). The role of movement in the recognition of famous faces. Memory & Cognition, 27(6), 974–985. 10.3758/BF03201228 10586574

[bibr50-20416695211054105] Lander K. Chuang L. (2005). Why are moving faces easier to recognize? Visual Cognition, 12(3), 429–442. 10.1080/13506280444000382

[bibr51-20416695211054105] Love B. C. Medin D. L. Gureckis T. M. (2004). SUSTAIN: A network model of category learning. Psychological Review, 111(2), 309–332. 10.1037/0033-295x.111.2.309 15065912

[bibr52-20416695211054105] Maechler M. Rousseeuw P. Struyf A. Hubert M. Hornik K. Studer M. , . . . Gonzalez J. (2016). Finding groups in data: Cluster analysis extended rousseeuw, Et al. R package version, 2(4).

[bibr53-20416695211054105] Matthews C. M. Davis E. E. Mondloch C. J. (2018). Getting to know you: The development of mechanisms underlying face learning. Journal of Experimental Child Psychology, 167, 295–313. 10.1016/j.jecp.2017.10.012 29220715

[bibr54-20416695211054105] Matts P. J. Fink B. (2010). Chronic sun damage and the perception of age, health and attractiveness. Photochemical & Photobiological Sciences, 9(4), 421–431. 10.1039/b9pp00166b 20354634

[bibr55-20416695211054105] Menon N. White D. Kemp R. I. (2015). Variation in photos of the same faced improvements in identity verification. Perception, 44(11), 1332–1341. 10.1177/0301006615599902 26562899

[bibr56-20416695211054105] Mileva M. Young A. W. Jenkins R. Burton A. M. (2020). Facial identity across the lifespan. Cognitive Psychology, 116, 101260. 10.1016/j.cogpsych.2019.101260 31865002

[bibr57-20416695211054105] Milligan G. W. (1980). An examination of the effect of six types of error perturbation on fifteen clustering algorithms. Psychometrika, 45(3), 325–342. 10.1007/bf02293907

[bibr58-20416695211054105] Murphy J. Ipser A. Gaigg S. B. Cook R. (2015). Exemplar variance supports robust learning of facial identity. Journal of Experimental Psychology: Human Perception and Performance, 41(3), 577–581. 10.1037/xhp0000049 25867504 PMC4445380

[bibr59-20416695211054105] Muth C. Carbon C. C. (2013). The aesthetic Aha: On the pleasure of having insights into gestalt. Acta Psychologica, 144(1), 25–30. 10.1016/j.actpsy.2013.05.001 23743342

[bibr60-20416695211054105] Neumann P. G. (1974). An attribute frequency model for the abstraction of prototypes. An attribute frequency model for the abstraction of prototypes, 2(2), 241–248. 10.3758/BF03208990 24214749

[bibr61-20416695211054105] Neumann P. G. (1977). Visual prototype formation with discontinuous representation of dimensions of variability. Memory & Cognition, 5(2), 187–197. 10.3758/BF03197361 24202810

[bibr62-20416695211054105] O'Toole A. J. Roark D. (2011). Memory of moving faces: The interplay of two recognition systems. In Curio C. Bülthoff H. H. Giese M. A. (Eds.), Dynamic faces: Insights from experiments and computation (1 ed., pp. 15–29). The MIT Press.

[bibr63-20416695211054105] Patterson K. Baddeley A. (1977). When face recognition fails. Journal of Experimental Psychology: Human Learning and Memory, 3(4), 406–417. 10.1037/0278-7393.3.4.406 864389

[bibr64-20416695211054105] Posner M. I. Keele S. W. (1968). On the genesis of abstract ideas. Journal of Experimental Psychology, 77(3), 353–363. 10.1037/h0025953 5665566

[bibr65-20416695211054105] Posner M. I. Keele S. W. (1970). Retention of abstract ideas. Journal of Experimental Psychology, 83(2, Pt.1), 304–308. 10.1037/h0028558 5665566

[bibr66-20416695211054105] R Core Team (2013). *R: A language and environment for statistical computing*. Retrieved from http://r.meteo.uni.wroc.pl/web/packages/dplR/vignettes/intro-dplR.pdf.

[bibr67-20416695211054105] Reed S. K. (1972). Pattern recognition and categorization. Cognitive Psychology, 3(3), 382–407. 10.1016/0010-0285(72)90014-X

[bibr68-20416695211054105] Reinitz M. T. Lammers W. J. Cochran B. P. (1992). Memory-conjunction errors: Miscombination of stored stimulus features can produce illusions of memory. Memory & Cognition, 20(1), 1–11. 10.3758/Bf03208247 1549060

[bibr69-20416695211054105] Ritchie K. L. Burton A. M. (2017). Learning faces from variability. The Quarterly Journal of Experimental Psychology, 70(5), 897–905. 10.1080/17470218.2015.1136656 26831280

[bibr70-20416695211054105] Ritchie K. L. Kramer R. S. S. Burton A. M. (2018). What makes a face photo a ‘good likeness’? Cognition, 170(Supplement C), 1–8. 10.1016/j.cognition.2017.09.001 28917125

[bibr71-20416695211054105] Schneider T. M. Carbon C. C. (2014). Why is this specific image of madonna the most prototypical one? Predicting prototypicality on basis of inspection frequency and familiarity. Perception, 43(S), 17–17.

[bibr72-20416695211054105] Schneider T. M. Carbon C. C. (2017a). How a face becomes familiar? Episodic facial prototypes and representations are generated across the life-span. Perception, 46(S), 34–34.

[bibr73-20416695211054105] Schneider T. M. Carbon C. C. (2017b). Taking the perfect selfie: Investigating the impact of perspective on the perception of higher cognitive variables. Frontiers in Psychology, 8(971), 1–16. 10.3389/fpsyg.2017.00971 28649219 PMC5465279

[bibr74-20416695211054105] Schneider T. M. Hecht H. Carbon C. C. (2012). Judging body weight from faces: The height-weight illusion. Perception, 41(1), 121–124. 10.1068/p7140 22611670

[bibr75-20416695211054105] Solso R. L. McCarthy J. E. (1981b). Prototype formation: Central tendency model vs. Attribute-frequency model. Bulletin of the Psychonomic Society, 17(1), 10–11. 10.3758/BF03333651

[bibr76-20416695211054105] Spratling M. W. (2016). Predictive coding as a model of cognition. Cognitive Processing, 17(3), 279–305. 10.1007/s10339-016-0765-6 27118562

[bibr77-20416695211054105] Strobach T. Carbon C. C. (2013). Face adaptation effects: Reviewing the impact of adapting information, time, and transfer. Frontiers in Psychology, 4, 1–12. 10.3389/fpsyg.2013.00318 23760550 PMC3669756

[bibr78-20416695211054105] Strobach T. Ditye T. Carbon C. C. (2011). Long-term adaptation effects of highly familiar faces are modulated by adaptation duration. Perception, 40(8), 1000–1004. 10.1068/p6986 22132514

[bibr79-20416695211054105] Tibshirani R. Walther G. Hastie T. (2001). Estimating the number of clusters in a data set via the gap statistic. Journal of the Royal Statistical Society Series B – Statistical Methodology, 63(1), 411–423. 10.1111/1467-9868.00293

[bibr80-20416695211054105] Tinio P. P. L. Leder H. (2009). Just how stable are stable aesthetic features? Symmetry, complexity, and the jaws of massive familiarization. Acta Psychologica, 130(3), 241–250. 10.1016/j.actpsy.2009.01.001 19217589

[bibr501-20416695211054105] Thorpe S. Fize D. Marlot C. (1996). Speed of processing in the human visual system. Nature, 381(6582), 520–522. 10.1016/j.actpsy.2009.01.001 8632824

[bibr81-20416695211054105] Valentine T. (1991). A unified account of the effects of distinctiveness, inversion, and race in face recognition. The Quarterly Journal of Experimental Psychology A: Human Experimental Psychology, 43(2), 161–204. 10.1080/14640749108400966 1866456

[bibr82-20416695211054105] Valentine T. Bruce V. (1986). The effects of distinctiveness in recognizing and classifying faces. Perception, 15(5), 525–535. 10.1068/p150525 3588212

[bibr83-20416695211054105] Valentine T. Endo M. (1992). Towards an exemplar model of face processing: The effects of race and distinctiveness. The Quarterly Journal of Experimental Psychology A: Human Experimental Psychology, 44(4), 671–703. 10.1080/14640749208401305 1615169

[bibr84-20416695211054105] Vanpaemel W. Storms G. (2008). In search of abstraction: The varying abstraction model of categorization. Psychonomic Bulletin & Review, 15(4), 732–749. 10.3758/Pbr.15.4.732 18792499

[bibr85-20416695211054105] Ward J. H. (1963). Hierarchical grouping to optimize an objective function. Journal of the American Statistical Association, 58(301), 236–244. 10.1080/01621459.1963.10500845

[bibr86-20416695211054105] Webster M. A. Kaping D. Mizokami Y. Duhamel P. (2004). Adaptation to natural facial categories. Nature, 428(6982), 557–561. 10.1038/Nature02420 15058304

[bibr87-20416695211054105] Webster M. A. MacLeod D. I. A. (2011). Visual adaptation and face perception. Philosophical Transactions of the Royal Society B-Biological Sciences, 366(1571), 1702–1725. 10.1098/Rstb.2010.0360 PMC3130378 21536555

[bibr88-20416695211054105] White D. Kemp R. I. Jenkins R. Burton A. M. (2014). Feedback training for facial image comparison. Psychonomic Bulletin & Review, 21(1), 100–106. 10.3758/s13423-013-0475-3 23835616

[bibr89-20416695211054105] Wickham H. (2016). Ggplot2: Elegant graphics for data analysis (2 ed.). Springer International Publishing.

[bibr90-20416695211054105] Wirth B. E. Carbon C. C. (2017). An easy game for frauds? Effects of professional experience and time pressure on passport-matching performance. Journal of Experimental Psychology: Applied, 23(2), 138–157. 10.1037/xap0000114 28368188

[bibr91-20416695211054105] Young A. W. Burton A. M. (2017). Recognizing faces. Current Directions in Psychological Science, 26(3), 212–217. 10.1177/0963721416688114

[bibr92-20416695211054105] Yu A. J. Dayan P. (2005). Uncertainty, neuromodulation, and attention. Neuron, 46(4), 681–692. 10.1016/j.neuron.2005.04.026 15944135

